# The function of glutaredoxin GRXS15 is required for lipoyl-dependent dehydrogenases in mitochondria

**DOI:** 10.1093/plphys/kiab172

**Published:** 2021-04-15

**Authors:** Anna Moseler, Inga Kruse, Andrew E Maclean, Luca Pedroletti, Marina Franceschetti, Stephan Wagner, Regina Wehler, Katrin Fischer-Schrader, Gernot Poschet, Markus Wirtz, Peter Dörmann, Tatjana M Hildebrandt, Rüdiger Hell, Markus Schwarzländer, Janneke Balk, Andreas J Meyer

**Affiliations:** 1 Institute of Crop Science and Resource Conservation (INRES)—Chemical Signalling, University of Bonn, 53113 Bonn, Germany; 2 Université de Lorraine, INRAE, IAM, Nancy 54000, France; 3 Department of Biological Chemistry, John Innes Centre, Norwich NR4 7UH, UK; 4 School of Biological Sciences, University of East Anglia, Norwich NR4 7TJ, UK; 5 Institute of Molecular Physiology and Biotechnology of Plants (IMBIO), University of Bonn, 53115 Bonn, Germany; 6 Department of Chemistry, Institute for Biochemistry, University of Cologne, 50674 Cologne, Germany; 7 Centre for Organismal Studies, University of Heidelberg, 69120 Heidelberg, Germany; 8 Institute of Plant Genetics, Leibniz University Hannover, 30167 Hannover, Germany; 9 Institute of Plant Biology and Biotechnology (IBBP)—Plant Energy Biology, University of Münster, 48143 Münster, Germany; 10 Bioeconomy Science Center, c/o Forschungszentrum Jülich, 52425 Jülich, Germany

## Abstract

Iron–sulfur (Fe–S) clusters are ubiquitous cofactors in all life and are used in a wide array of diverse biological processes, including electron transfer chains and several metabolic pathways. Biosynthesis machineries for Fe–S clusters exist in plastids, the cytosol, and mitochondria. A single monothiol glutaredoxin (GRX) is involved in Fe–S cluster assembly in mitochondria of yeast and mammals. In plants, the role of the mitochondrial homolog GRXS15 has only partially been characterized. Arabidopsis (*Arabidopsis thaliana*) *grxs15* null mutants are not viable, but mutants complemented with the variant *GRXS15 K83A* develop with a dwarf phenotype similar to the knockdown line *GRXS15^amiR^*. In an in-depth metabolic analysis of the variant and knockdown *GRXS15* lines, we show that most Fe–S cluster-dependent processes are not affected, including biotin biosynthesis, molybdenum cofactor biosynthesis, the electron transport chain, and aconitase in the tricarboxylic acid (TCA) cycle. Instead, we observed an increase in most TCA cycle intermediates and amino acids, especially pyruvate, glycine, and branched-chain amino acids (BCAAs). Additionally, we found an accumulation of branched-chain α-keto acids (BCKAs), the first degradation products resulting from transamination of BCAAs. In wild-type plants, pyruvate, glycine, and BCKAs are all metabolized through decarboxylation by mitochondrial lipoyl cofactor (LC)-dependent dehydrogenase complexes. These enzyme complexes are very abundant, comprising a major sink for LC. Because biosynthesis of LC depends on continuous Fe–S cluster supply to lipoyl synthase, this could explain why LC-dependent processes are most sensitive to restricted Fe–S supply in *grxs15* mutants.

## Teaser

Decreased activity of the mitochondrial glutaredoxin GRXS15 causes metabolic defects associated with impaired lipoyl cofactor-dependent dehydrogenase complexes.

## Introduction

Since the early days of biological evolution, iron–sulfur (Fe–S) clusters have been employed as catalytic co-factors for electron transfer reactions and are nowadays present in a plethora of essential proteins (Pain and Dancis, 2016). Because Fe–S clusters are inherently unstable they do not exist in free form but always need to be chaperoned before reaching their final destination apoproteins. Among the proteins thought to be involved in Fe–S cluster transfer is a specific subtype of glutaredoxins (GRXs) capable of coordinating [2Fe–2S] clusters as a protein dimer (Banci et al., 2014; Couturier et al., 2015; Lill and Freibert, 2020).

GRXs are ubiquitous proteins, which form a large family with several subfamilies in plants (Rouhier et al., 2008; Meyer et al., 2009). Although their canonical function is glutathione-dependent redox catalysis, dissection of the function of subclasses and individual family members reveals an unexpectedly diverse picture (Deponte, 2013; Liedgens et al., 2020; Trnka et al., 2020). Class II GRXs share a CGFS amino acid motif in the active site and are proposed to serve as carrier proteins for Fe–S cluster between the assembly machinery and receiving apoproteins. A second proposed function is the repair of oxidation-sensitive Fe–S clusters (Couturier et al., 2015). In Arabidopsis (*Arabidopsis thaliana*), Fe–S cluster assembly machineries are present in the cytosol, plastids, and mitochondria, and at least one monothiol GRX is located in each of these compartments: GRXS15 in mitochondria; GRXS14 and GRXS16 in plastids; and GRXS17 in the cytosol (Cheng et al., 2006; Bandyopadhyay et al., 2008; Knuesting et al., 2015; Moseler et al., 2015). While plants deficient in plastidic GRXS14 did not display any growth phenotype under nonstress conditions, genetic stacking of a *grxs14* null mutant and knockdown of *GRXS16* caused pronounced growth retardation (Rey et al., 2017). Exposure of *grxs14* and the double mutant to prolonged darkness led to accelerated chlorophyll loss compared to wild-type (WT) and decreased abundance of proteins involved in the maturation of Fe–S proteins. Mutants lacking the cytosolic GRXS17 were sensitive to high temperature and long-day photoperiod (Cheng et al., 2011; Knuesting et al., 2015; Martins et al., 2020). However, the activities of cytosolic Fe–S proteins, like aconitase (ACO) or aldehyde oxidase (AO), were not substantially altered in *grxs17* null mutants (Knuesting et al., 2015; Iñigo et al., 2016).

The mitochondrial GRXS15 is indispensable as indicated by embryonic lethality of null mutants (Moseler et al., 2015). Partial complementation with a mutated *GRXS15 K83A* variant, which is weakened in its ability to coordinate an [2Fe–2S] cluster in vitro, results in a dwarf phenotype (Moseler et al., 2015). A similar dwarf phenotype has also been reported for a *GRXS15* knockdown line (Ströher et al., 2016). Expression of *GRXS15* in the yeast (*Saccharomyces cerevisiae*) *grx5* mutant can partially rescue the impaired growth and ACO activity (Moseler et al., 2015), suggesting a conserved function in Fe–S cluster assembly. Grx5 in yeast and its homolog in vertebrates are thought to have a general role in the early steps of Fe–S cluster transfer leading to pleiotropic effects due to the simultaneous impairment of several central mitochondrial processes (Lill and Freibert, 2020). In vitro data showed that Arabidopsis GRXS15 was able to interact with the scaffold proteins iron-sulfur cluster assembly (ISCA)1a/2 and to deliver [2Fe–2S] cluster resulting in the generation of [4Fe–4S] cluster on the ISCA complex (Azam et al., 2020). Mitochondria contain at least 26 Fe–S proteins that are involved in different processes, including electron transport [complexes I–III in the respiratory electron transport chain (ETC)] and the tricarboxylic acid (TCA) cycle [ACO and succinate dehydrogenase (SDH)]. A general role of GRXS15 in the early steps of Fe–S cluster transfer would therefore predict pleiotropic effects of diminished GRXS15 activity, due to the simultaneous impairment of several central mitochondrial processes. The number of potential defective sites is even further amplified if the synthesis of enzyme cofactors and the function of several cofactor-dependent enzymes, in turn, is compromised. Indeed, pathways for biosynthesis of the molybdenum cofactor (Moco) and lipoyl cofactor (LC) involve the mitochondrial [4Fe–4S] proteins GTP-3′,8-cyclase (CNX2; cofactor of nitrate reductase [NR] and xanthine dehydrogenase [XDH] 2) and lipoyl synthase (LIP1; Yasuno and Wada, 2002; Schwarz and Mendel, 2006). It should be noted that *Δgrx5* mutants in yeast also have a defect in iron signaling and accumulate toxic levels of iron as a consequence (Rodríguez-Manzaneque et al., 2002), for which there is so far no evidence in Arabidopsis *grxs15* mutants.

A study of two different *GRXS15* knockdown mutants in Arabidopsis by Ströher et al. (2016) found normal activities of ACO (one [4Fe-4S] cluster) and complex I (one [2Fe-2S]cluster and seven [4Fe-4S] clusters) but a pronounced decrease in LC-dependent proteins. Diminished presence of the LC itself and decreased abundance of mitochondrial LIP1 (mLIP1) led to the conclusion that GRXS15 plays a putative role in transfer of Fe–S clusters for mitochondrial LC synthesis (Ströher et al., 2016). In the mitochondrial matrix, four enzyme complexes depend on lipoamide as a prosthetic group: the pyruvate dehydrogenase complex (PDC), the 2-oxoglutarate (2-OG) dehydrogenase complex (OGDC), the glycine decarboxylase complex (GDC), and the branched-chain α-keto acid (BCKA) dehydrogenase complex (BCKDC; Taylor et al., 2004; Solmonson and DeBerardinis, 2018). The PDC acts as the entry point of acetyl-CoA into the TCA cycle, while OGDC acts within the TCA cycle to convert 2-OG to succinyl-CoA. The GDC catalyzing the oxidative decarboxylation of glycine is essential for photorespiration (Douce et al., 2001), but also for C1 metabolism (Mouillon et al., 1999). BCKDC is involved in the catabolism of the three branched-chain amino acids (BCAAs) leucine (Leu), valine (Val), and isoleucine (Ile), and their corresponding BCKAs (Araújo et al., 2010; Gu et al., 2010; Peng et al., 2015). Whether all these LC-dependent enzymes are affected similarly in *grxs15* mutants and whether other pathways containing Fe–S enzymes are diminished and thus constitute bottlenecks that severely restrict metabolic fluxes is yet unknown because the respective mutants have not been metabolically characterized.

Here, we aimed to identify the most severe metabolic bottlenecks caused by severely restricted capacity of GRXS15 mutants in Fe–S transfer. We consider several candidate Fe–S proteins involved in essential mitochondrial processes starting with biotin biosynthesis, followed by Moco biosynthesis, capacity of the mitochondrial ETC (mETC), TCA cycle flow, and closing with the biosynthesis of LC. We assess how these Fe–S-related processes are affected in *grxs15-3* null mutants complemented with *GRXS15 K83A* and in *GRXS15^amiR^* knockdown mutants trying to pin down the cause of the phenotype and by that the functional significance of GRXS15. By direct comparison of partially complemented null mutants and knockdown mutants, we resolve previous contradictions about the role of GRXS15 in the maturation of Fe–S containing enzymes.

## Results

### *GRXS15 K83A* causes retardation in growth

**Figure 1:**
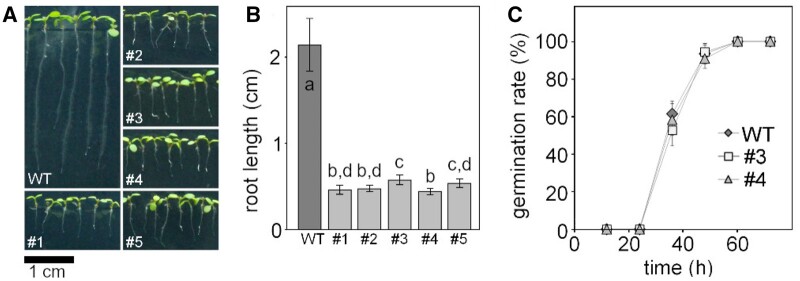
Complementation of the Arabidopsis *grxs15-3* mutant with *UBQ10_pro_:GRXS15 K83A*. A, 8-d-old WT seedlings compared with *GRXS15 K83A* mutants grown on vertical agar plates under long-day conditions. B, Primary root length of 8-d-old *GRXS15 K83A* mutants compared to WT (*n* = 35; means ± sd). Different letters indicate significant differences between the different lines; *P* ≤ 0.05; (one-way analysis of variance (ANOVA) with post hoc Holm–Sidak analyses ). C, Germination rate of *GRXS15 K83A* lines #3 and #4 compared to WT. All seeds were initially stratified at 4°C in the dark for 1 d (*n* = 6 with 20–25 seeds each; means ± sd). Germination was assessed with the emergence of the radicle. No statistically significant differences were found using Student’s *t* test analysis (*P* > 0.05).

To complete embryogenesis, GRXS15 is essential in plants. To bypass embryo lethality, Arabidopsis *grxs15* null mutants were complemented with the *GRXS15 K83A* variant and these complemented plants are able to grow, but have small rosette leaves (Moseler et al., 2015). Based on this observation, we aimed to further analyze the growth phenotype and compare it with published records of *grxs15* knockdown mutants. The dwarf phenotype of the *GRXS15 K83A* complementation lines #1 to #5 becomes apparent at the early seedling stage (Figure 1, A and B). Analysis of root length in five randomly selected lines consistently also showed a concomitant reduction of primary root length compared to WT (Figure 1B). Although only minor differences in seedling size could be observed, line #3 was the best growing complementation line, and line #4 the weakest (Figure 1C; Moseler *et al.*, 2015). This effect was stable and consistent over several generations. The phenotype is similar to *GRXS15^amiR^* knockdown lines reported by (Ströher et al. 2016; Supplemental Figure S1). The insertion line *grxs15-1* carrying a T-DNA in an intron within the 5′-UTR (Moseler et al. 2015), which had been reported to display a short root phenotype (Ströher et al., 2016) cannot be clearly distinguished from the WT in our hands, neither at seedling stage nor at rosette stage (Supplemental Figure S1). This allele was excluded from further analysis. To test whether the reduced growth of *GRXS15 K83A*-complemented null mutants was true growth retardation or caused by delayed germination, the two lines #3 and #4 were scored for the timing of radical emergence. The absence of any difference between WT and the two mutants suggests that the growth phenotype reflects a genuine growth retardation (Figure 1C).

### Biotin-mediated metabolism is not impaired when GRXS15 function is diminished

**Figure 2:**
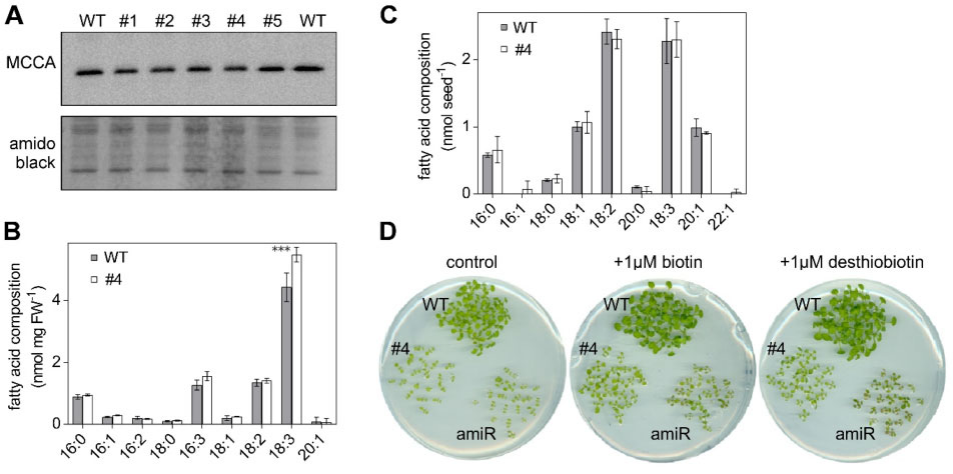
*GRXS15 K83A* mutation has no impact on the biotin pathway in Arabidopsis seedlings. A, Immunoblot analysis of biotinylated MCCA in mitochondria (mito) of *GRXS15 K83A* mutants compared with WT. In the upper, biotinylated MCCA was detected by streptavidin HRP in isolated mito from 2-week-old seedlings (9 μg protein was loaded per lane). In the lower, amido black staining of the membrane is shown as a control for protein loading. B and C, Fatty acids quantified by gas chromatography of 8-d-old seedlings (B) and seeds (C) of *GRXS15 K83A* line #4 compared to WT (*n* = 3–4; means ± sd). The statistical analysis (two-way ANOVA with post hoc Holm–Sidak comparisons for WT versus *grxs15*) indicated no significant (*P* ≤ 0.05) change except for 18:3 (***P < 0.001). D, *GRXS15 K83A* line #4, the knockdown line *GRXS15^amiR^* (amiR), and WT plants were grown on horizontal plates with 1/2 MS agar without sucrose. The medium contained either no biotin (control), 1 μM biotin or 1 μM desthiobiotin.

Following our earlier observation that GRXS15 can coordinate a [2Fe–2S] cluster (Moseler et al., 2015), similar to the homologs in yeast and mammals (Uzarska et al., 2013; Banci et al., 2014), we embarked on testing a number of pathways of Fe–S-dependent metabolism that may be affected in the mutant. One putative target protein of GRXS15 is mitochondrial biotin synthase (BIO2, At2g43360) since it relies on supply of a [2Fe–2S] and a [4Fe–4S] cluster. BIO2 catalyzes the final step in biotin biosynthesis, which acts as an essential cofactor in several carboxylases in energy metabolism. Destruction of the [2Fe–2S] cluster for sulfur supply to biotin with each catalytic cycle and subsequent turnover increases the demand of the BIO2 protein for [2Fe–2S] clusters (Ugulava et al., 2001). *bio2* null mutants were previously described as embryo-defective, arrested mostly at globular or heart stage of embryo development (Patton et al., 1998; Meinke, 2019). Because lack of biotin typically causes degradation of the respective apoproteins (Solbiati et al., 2002), we tested for the abundance of biotin-dependent methylcrotonoyl-CoA carboxylase (MCCase), which is involved in Leu degradation in mitochondria. None of the five analyzed *grxs15* complementation lines showed a decrease in protein abundance of the biotinylated MCCase subunit A (MCCA; Figure 2A). Biotin is also exported to the cytosol and the chloroplasts, where it is required for synthesis and elongation of fatty acids by hetero and homomeric acetyl-CoA carboxylase (ACCase). Total fatty acids in seeds amounted to 7.6 ± 0.8 nmol seed^−1^ in line #4 and 7.6 ± 1.0 nmol seed^−1^ in the WT and no difference in relative abundance of specific fatty acids in seeds was observed (Figure 2C). In 8-d-old seedlings, the amount of total fatty acids did not differ in line #4 10.3 ± 0.4 nmol (mg FW)^−1^ compared to 8.8 ± 1.0 nmol (mg FW)^−1^ in WT, but a 23% increase in α-linolenic acid (18:3) was observed (Figure 2B).

*bio2* mutants can be rescued by the addition of biotin to both arrested embryos cultured in vitro and to mutant plants grown on soil (Schneider et al., 1989; Patton et al., 1998; Pommerrenig et al., 2013). External supply of biotin or its precursor desthiobiotin to a *GRXS15^amiR^* knockdown mutant and the complemented line #4 in both cases improved growth slightly but did not rescue the growth defects of either of the lines (Figure 2D). It should be noted though that also the WT grew better with the supply of biotin or desthiobiotin. These results suggest that growth retardation of *grxs15* mutants is not primarily caused by defects in biotin synthesis.

### Moco-dependent nitrogen metabolism is not limiting upon impaired GRXS15 function

**Figure 3:**
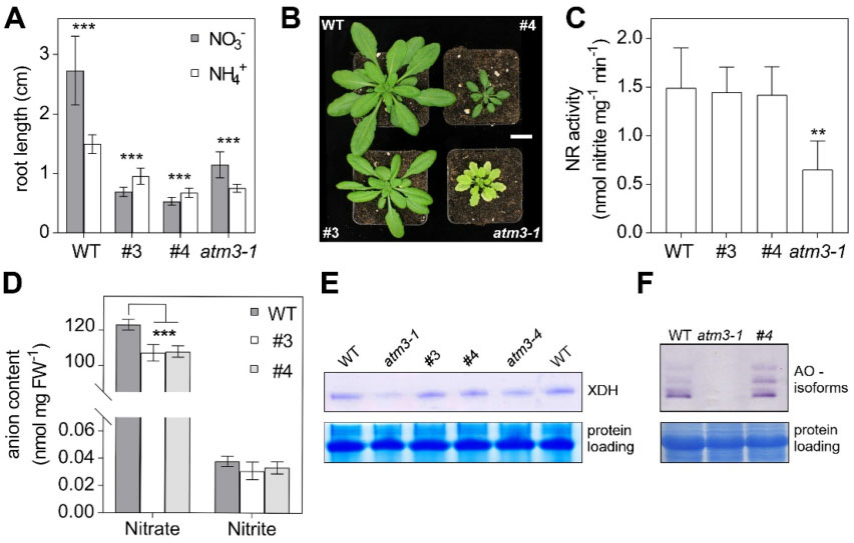
Growth of Arabidopsis *GRXS15 K83A* mutants is affected by the nitrogen source. A, Primary root length of *GRXS15 K83A* lines #3 and #4 as well as *atm3-1* seedlings compared to WT grown on vertical agar plates containing 5 mM KNO_3_ or 2.5 mM (NH_4_)_2_SO_4_ as N-source for 8 d under long-day conditions (*n* = 30; means ± sd). Student’s *t* test analysis showed significant differences between the growth on the different inorganic N-sources in all lines ***P < 0.001. B, Representative 4-week-old plants of WT, *GRXS15 K83A* lines #3 and #4, and *atm3-1* all grown on soil under long-day conditions. Scale bar = 2 cm. C, NR activity in WT, lines #3 and #4 as well as in *atm3-1*. Activity was analyzed in 4-week-old plants grown on soil by measuring the presence of nitrite via the Griess reaction (*n* = 4; means ± sd, one-way ANOVA with post hoc Holm–Sidak comparisons for WT versus mutant lines **P ≤ 0.01). D, Nitrate and nitrite content of 8-d-old WT and *GRXS15 K83A* lines #3 and #4 seedlings grown on agar plates (*n* = 4; means ± sem). The statistical analysis (two-way ANOVA with post hoc Holm–Sidak comparisons for WT versus *grxs15*) indicated a significant change in the nitrate content; ***P ≤ 0.001. E, In-gel activity of XDH in WT, *atm3-1*, and *GRXS15 K83A* mutants. Equal amounts of protein (35 μg) extracted from 8-d-old seedlings were separated on nondenaturing PA gel and stained for XDH activity using hypoxanthine as substrate. As a control of protein-loading, the gel was subsequently stained with Coomassie. F, In-gel activities of AO in WT and *atm3-1* as well as *grxs15* mutants. Equal amounts of protein were separated by nondenaturing PAGE and stained for AO activity using synthetic aldehydes (1-naphthaldehyde and indole-3-carboxaldehyde) as substrates. For control of protein loading, the gel was subsequently stained with Coomassie.

The Moco precursor cyclic pyranopterin monophosphate (cPMP) is synthesized in the mitochondrial matrix by the CNX2 (At2g31955) and the cPMP synthase CNX3 (At1g01290) and is exported to the cytosol for subsequent biosynthesis steps (Bittner, 2014; Kruse et al., 2018). Because CNX2 contains two [4Fe–4S] clusters, we hypothesized that Moco biosynthesis and hence Moco-dependent biochemical pathways may be affected by defects in mitochondrial Fe–S transfer. The most abundant Moco-dependent enzymes include NR, AO, XDH, and sulfite oxidase. Arabidopsis generally prefers nitrate as nitrogen source (Sarasketa et al., 2014), but mutants deficient in Moco biosynthesis can be rescued by providing ammonium as a nitrogen source to bypass NR (Wang et al., 2004; Kruse et al., 2018), revealing NR as the main recipient of Moco. While the preference for nitrate (KNO_3_) over ammonium ((NH_4_)_2_SO_4_) could be confirmed in WT plants, we found that the growth retardation of *GRXS15 K83A* roots is more pronounced on nitrate than on ammonium as sole nitrogen source (Figure 3A). Similar results were obtained when seedlings were grown on NH_4_Cl instead of (NH_4_)_2_SO_4_ to control for possible impacts of the respective counter anions on the growth behavior (Supplemental Figure S2A).

The pronounced growth retardation on nitrate could be indicative of severe NR deficiency similar to *nia1 nia2* mutants lacking functional NR (Wilkinson and Crawford, 1993). A similar NR deficiency has been described for mutant alleles of the ABC transporter ATM3 that is involved in Moco biosynthesis (Bernard et al., 2009; Teschner et al., 2010; Kruse et al., 2018). *atm3-1* mutants display a severe growth phenotype and are chlorotic (Figure 3B). While *GRXS15 K83A* mutants are also smaller than WT, they are not chlorotic and thus do not phenocopy *atm3-1* (Figure 3, A and B). Despite NR activity being diminished to 50% of WT, root growth of *atm3-1* was still better on nitrate than on ammonium (Figure 3, A and C). NR activity was not altered in the *GRXS15 K83A* mutants #3 and #4 (Figure 3C). Despite the unaffected NR activity, both *grxs15* mutants contained significantly less nitrate than WT seedlings (Figure 3D). Nitrite and other inorganic anions like chloride, sulfate, or phosphate were not altered between the mutant lines and WT (Supplemental Figure S2B). All other tested Moco-dependent enzymes such as AO or XDH showed no decrease in activity in the *grxs15* mutants compared to WT (Figure 3, E and F). Taken together, these results suggest that NR activity in *GRXS15 K83A* mutants is sufficient to use nitrate as the sole nitrogen source and does not explain the growth inhibition on nitrate.

### Impaired GRXS15 function leads to decreased root respiration

**Figure 4:**
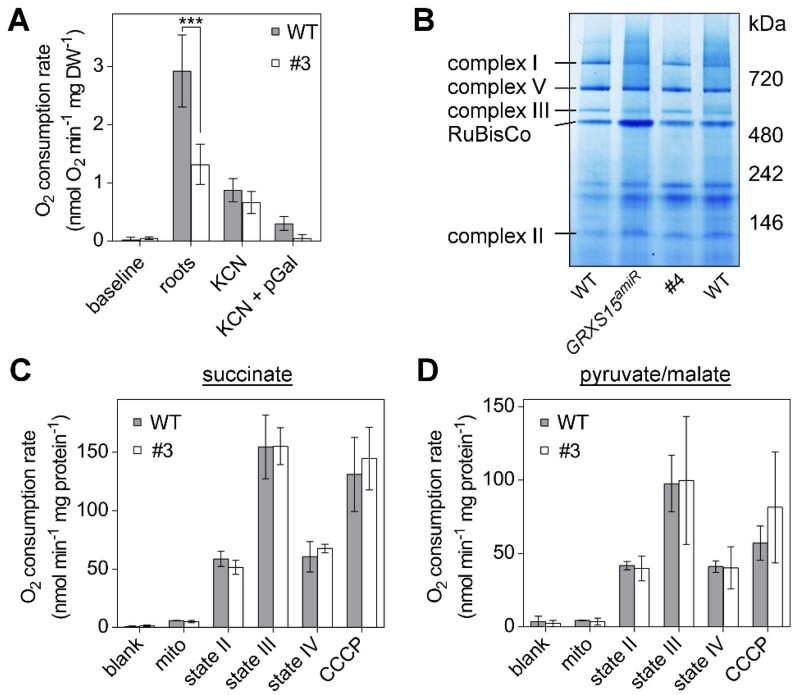
Respiration in complemented Arabidopsis *grxs15* mutants. A, Root respiration rate of *GRXS15 K83A* line #3 (4.5-week old) and the respective WT grown to similar size (2-week old) after addition of the cytochrome c oxidase inhibitor KCN (4 mM) alone or together with the AOX inhibitor pGal (0.2 mM; *n* = 4; means ± sd). The statistical analysis (two-way ANOVA with post hoc Holm–Sidak comparisons for WT versus *grxs15* mutant) indicated a significant difference in the respiration of mito from WT and *GRXS15 K83A* line #3; ***P ≤ 0.001. B, Respiratory complexes I, II, III, and V separated by Blue Native–PAGE and visualized with Coomassie staining in WT, *GRXS15 K83A* line #4, and *GRXS15^amiR^*. Mito were purified from 4-week-old plants. C and D, O_2_ consumption rates for purified mito from WT *and GRXS15 K83A* line #3 energized with succinate (left) or pyruvate/malate (right). O_2_ consumption was measured before (blank) and after addition of mito. State II respiration was initiated by the addition of the respective substrate (State II; succinate (10 mM succinate, 0.25 mM ATP) or pyruvate/malate (10 mM pyruvate, 10 mM malate, 0.3 mM NAD, and 0.1 mM thiamine pyrophosphate). State III respiration was initiated by the addition of 50 μM ADP. State IV represents the respiration after ADP consumption and CCCP shows the respiration after addition of the protonophore carbonyl cyanide m-CCCP (10 μM), which uncouples electron transport from ATP synthesis. All results are based on three independent preparations of mito and are shown as means ± SEM.

The mETC contains three enzyme complexes with a total of 12 Fe–S cofactors: complex I with two [2Fe–2S] and six [4Fe–4S] clusters, complex II with one [2Fe–2S], one [3Fe–4S], and one [4Fe–4S] cluster, and complex III with one [2Fe–2S] cluster (Couturier et al., 2015; Meyer et al., 2019). Thus, we measured the respiration of detached roots and dissected the capacity of complexes I and II-linked electron flow. Indeed, roots of line #3 displayed a decreased respiration rate of 1.31 ± 0.35 nmol oxygen (O_2_) min^−1^ (mg DW)^−1^ compared with the WT rate of 2.92 ± 0.62 nmol O_2_ min^−1^ (mg DW)^−1^ (Figure 4A). This is similar to root tips of *GRXS15^amiR^* knockdown plants, which were reported to consume less O_2_ than WT plants (Ströher et al., 2016). Addition of the cytochrome *c* oxidase inhibitor KCN decreased the rate of both lines down to similar values. The remaining rates are accounted for by the presence of alternative oxidases (AOXs), since they could be inhibited by propylgallate (pGal). Interestingly, the AOX capacity appeared unchanged in line #3, even though AOX is highly inducible by mitochondrial dysfunction. Next, we investigated if the decreased root respiration is due to defects in the respiratory machinery or due to restricted metabolite turnover, or both. First, we compared the abundance of respiratory complexes in isolated mitochondria from *GRXS15 K83A* line #4, *GRXS15^amiR^* by Blue Native–PAGE. None of the respiratory complexes including the Fe–S cluster containing complexes I–III was decreased in abundance in either mutant (Figure 4B). Additionally, we purified mitochondria from whole seedlings of the *GRXS15 K83A* line #3 and supplemented them with succinate or pyruvate/malate, respectively, as respiratory substrates. Succinate provides electrons to the ubiquinone pool of the mETC via complex II, while pyruvate/malate feeding predominantly provides NADH mainly generated by malate dehydrogenase, NAD-malic enzyme, and PDC. In addition, NADPH is also generated mainly due to the promiscuous specificity of mitochondrial malate dehydrogenase, NAD-malic enzyme, which also accepts NADP^+^ as electron acceptor, and most prominently by NADP-isocitrate dehydrogenase (IDH; Møller and Rasmusson, 1998; Møller et al., 2020). In addition to oxidation by complex I, NADH is also oxidized by NDA-type alternative dehydrogenases (Elhafez et al., 2006). NADPH is oxidized by NDC-type alternative dehydrogenase, NADPH-dependent thioredoxin reductases A and B, and glutathione reductase 2 (Reichheld et al., 2005; Rasmusson et al., 2008; Marty et al., 2019). No differences in the respiration of isolated mitochondria were found with supply of succinate or pyruvate/malate (Figure 4, C and D), suggesting that the differences in respiration observed in whole roots cannot be accounted for by decreased capacities of the Fe–S cluster-containing complexes. In summary, similar total respiratory activities of WT and mutants further indicate that the in vivo difference in respiration rate is not due to a defect at the level of the mETC, but rather upstream or downstream.

The capacity for electron flow in isolated mitochondria does not allow conclusions about the actual mETC activity in planta. Hence, we tested whether the decreased respiration rate may result in a change of the ATP status of the cells. For analyses of the MgATP^2−^ level WT plants as well as the *grxs15* mutants #3 and #4 were transformed with the MgATP^2−^ biosensor ATeam1.03-nD/nA (De Col et al., 2017) targeted to the cytosol. As cytosolic ATP is predominantly provided by the mitochondria (Igamberdiev et al., 2001; Voon et al., 2018), any disturbance in the mitochondrial ATP synthesis will also affect the ATP level in the cytosol. Similar to the report by De Col et al. (2017) higher Venus/cyan fluorescent protein (CFP) fluorescence ratios indicating more efficient Förster resonance energy transfer between the sensor subunits and hence higher MgATP^2−^ levels were found in cotyledons compared to roots (Supplemental Figure S3). However, no differences in the Venus/CFP emission ratio could be observed between WT and *GRXS15 K83A* mutants indicating similar cytosolic ATP levels (Supplemental Figure S3). It should be noted though that the energy charge of the adenylate pool cannot be deduced from these results as it would require also analysis of AMP and ADP.

**Figure 5:**
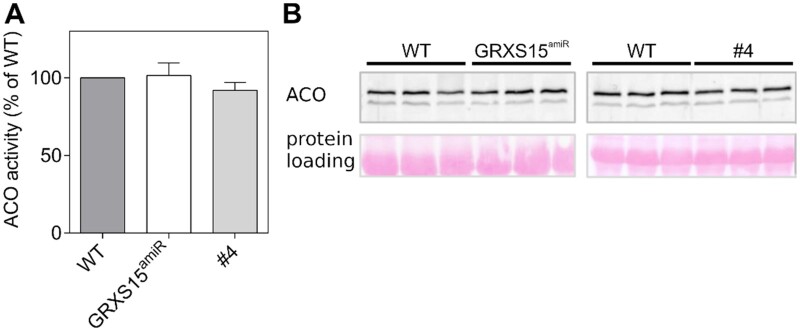
ACO activities in leaf extracts of *grxs15* mutants. A, ACO activity of *GRXS15^amiR^* and G*RXS15 K83A* line #4 compared to the respective WTs in leaf extracts from 3-week-old seedlings, measured by spectrophotometry in a coupled assay. *n* = 3; means ± sd. WT ACO activity was 35.3 ± 2.2 mU/mg protein for *GRXS15^amiR^* and 50.6 ± 5.1 mU/mg for the parental line of *GRXS15 K83A* lines, while the activity for the mutants was 35 ± 2.4 and 46.6 ± 2.1 mU/mg, respectively. B, Protein blot analysis of ACO in total leaf extracts from individual plants under denaturing conditions (all three ACO isozymes have the same electrophoretic mobility). Twenty micrograms of total protein extract was loaded. Total protein staining on the membrane after transfer with Ponceau S served as a loading control.

Previously, we reported a 60% decrease in ACO activity in *GRXS15 K83A* mutants while the total amount of ACO protein remained unchanged (Moseler et al., 2015). A decrease in ACO activity could at last partially explain the decreased respiration rate, but a decrease in ACO activity was not seen by Ströher et al. (2016). To clarify the situation, we measured the activity of ACO, a [4Fe–4S] enzyme, in the *GRXS15 K83A* and *GRXS15^amiR^* mutants grown side by side under the same conditions. We found no significant difference as a result of depleted or mutated GRXS15 protein in total leaf extracts (Figure 5A). Additionally, the abundance of ACO protein was the same (Figure 5B).

Furthermore, the mitochondrial ACO activity in purified mitochondria was analyzed in both *grxs15* mutant lines. Here, we found a slight decrease in ACO activity (normalized for citrate synthase) and no obvious difference in protein abundance (Supplemental Figure S4, A and B). However, we discovered that ACO is prone to partial inhibition in leaf extracts of the *grxs15* mutants, which likely caused the initially reported decrease (Supplemental Figure S4, C and D). The mutant-specific inhibition is not seen in isolated mitochondria; therefore, we argue that the decreased activity is due to the accumulation of a metabolite in the tissue that is removed in the course of the mitochondrial isolation.

### Diminished GRXS15 activity does not lead to any major signs of oxidative stress

Yeast *Δgrx5* mutant, as well as a Arabidopsis *grxs14* null mutant, are sensitive to oxidative stress and at least for the *Δgrx5*, it was shown that specific proteins are oxidized in this mutant (Rodríguez-Manzaneque et al., 1999; Cheng et al., 2006). Therefore, we analyzed the *GRXS15 K83A* mutant for any signs of oxidative stress that may result from iron-mediated reactive oxygen species (ROS) formation as a possible consequence of an improper Fe–S cluster transfer by the GRXS15 K83A protein variant. However, staining of leaves with 3, 3-diaminobenzidine (DAB) for H_2_O_2_ and nitro blue tetrazolium (NBT) for superoxide revealed no differences between WT and *grxs15* mutants (Supplemental Figure S5). Since histological stains only provide a crude indication of major changes in ROS dynamics, but are not sufficiently sensitive to resolve localized intracellular changes in oxidant load, we next analyzed mitochondria-specific changes in H_2_O_2_ concentration or the glutathione redox potential (*E*_GSH_). The genetically encoded sensors roGFP2-Orp1 (Nietzel et al., 2019) and roGFP2-hGrx1 (Albrecht et al., 2014) were expressed in the mitochondrial matrix of both WT and mutant plants. Both sensors were highly reduced under control conditions and neither roGFP2-Orp1 nor roGFP2-hGrx1 revealed any significant differences between WT and *GRXS15 K83A* mutants in mitochondria of cotyledons and root tips (Supplemental Figure S5, B and C). Both roGFP2-sensor variants remained highly reduced in all lines as indicated by similar fluorescence ratios that resembled those after incubation with dithiothreitol (DTT) for full sensor reduction. This indicates no major oxidative challenge in the mitochondrial matrix. Both sensors were responsive to oxidative challenge as indicated by a pronounced ratio change upon H_2_O_2_ addition.

### Diminished GRXS15 activity leads to accumulation of TCA cycle intermediates

**Figure 6:**
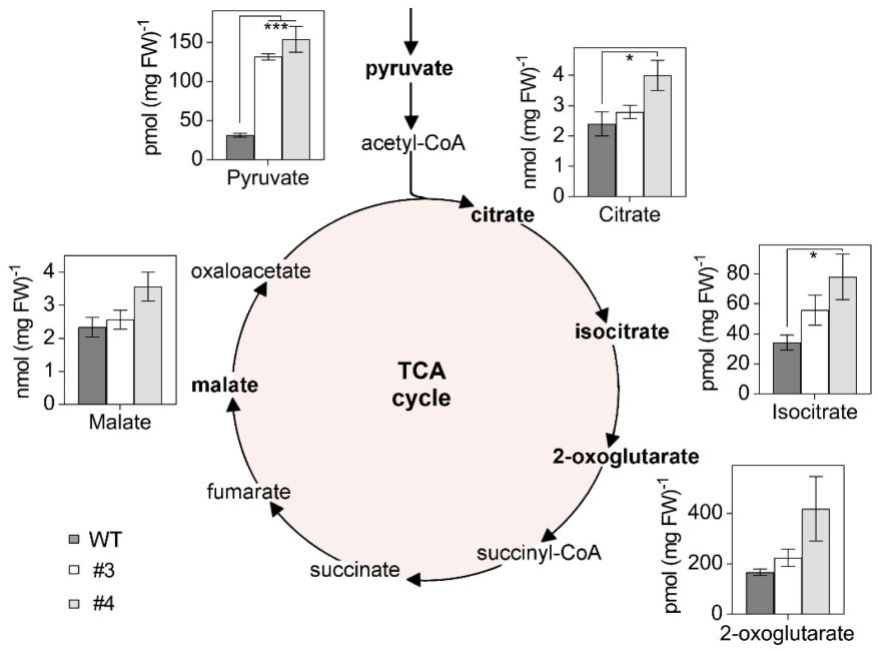
Organic acids of the TCA cycle accumulate in Arabidopsis *GRXS15 K83A* mutants. Organic acids were analyzed in 8-d-old seedlings of WT compared to *GRXS15 K83A* lines #3 and #4 (*n* = 4–5; means ± sem). The statistical analysis (one-way ANOVA with post hoc Holm–Sidak comparisons for WT vs. mutant lines) indicated significant changes; *P ≤ 0.05; ***P ≤ 0.001.

To investigate any other metabolic defects in the *GRXS15 K83A* mutant, we measured the concentrations of several organic acids in the *GRXS15 K83A* mutants. We found most of the analyzed organic acids in the complemented *grxs15* mutants #3 and #4 to be increased. Pyruvate showed the most pronounced change, increasing by more than four-fold from 31.5 ± 2.4 pmol (mg FW)^−1^ in the WT to 131.76 ± 3.8 and 153.97 ± 16.5 pmol (mg FW)^−1^ in lines #3 and #4 (Figure 6). The accumulation of citrate and isocitrate was significant in line #4, but not in line #3. 2-OG and malate showed minor increases in line #3 and pronounced increases in line #4. This trend did not reach statistical significance, however. A similarly concerted accumulation of TCA cycle intermediates was previously observed in antisense lines of the mitochondrial manganese superoxide dismutase 1 (MSD1; Morgan et al., 2008). Those lines showed impaired mitochondrial ACO activity to ˂50%, suggesting that the compromised ACO activity is sufficient as an explanation for the rearrangements in the pools of TCA cycle intermediates. However, pyruvate content was not determined in the *MSD1* antisense lines and the increased pyruvate content found in *GRXS15 K83A* lines cannot be straightforwardly linked to ACO activity.

### Alterations in pyruvate and glycine metabolism are associated with impairment of LC-dependent enzymes under diminished GRXS15 activity

**Figure 7:**
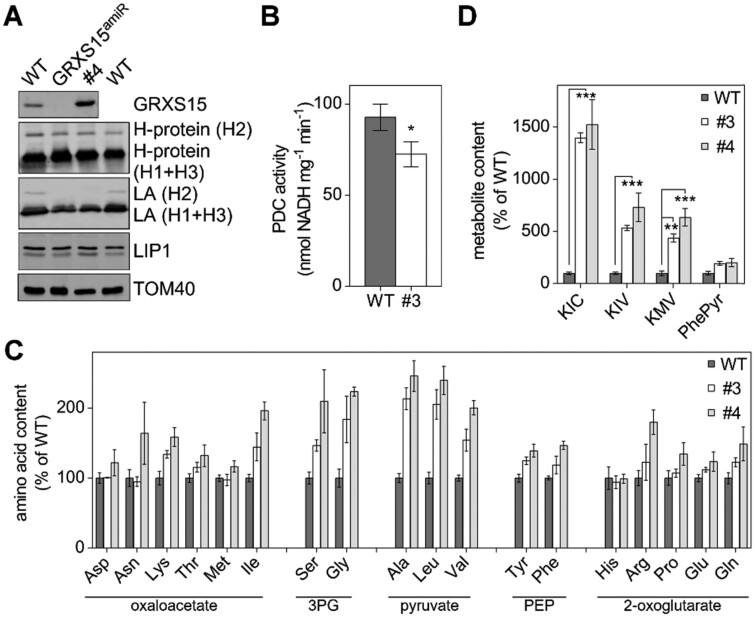
LC-dependent enzymes are affected in Arabidopsis *GRXS15 K83A* mutants. A, Immunoblot analysis using antibodies against GRXS15, glycine dehydrogenase H-protein (H1-3), LC (LA), mLIP1 as well as TOM40 for a loading control. Fifteen micrograms of isolated mitochondrial protein were loaded per lane. B, PDC activity in isolated mito. Reduction of NAD^+^ was measured in extracts of mito isolated from 14-d-old WT seedlings and the *GRXS15 K83A* line #3 (*n* = 5; means ± sem). The statistical analysis (one-way ANOVA with post hoc Holm–Sidak comparisons for WT versus *grxs15* mutant) indicated a significant change; *P = 0.025). C, Relative abundance of amino acids in 8-d-old seedlings of WT compared to *GRXS15 K83A* lines #3 and #4. WT was set to 100% (*n* = 4–5, means ± sem). Absolute values and statistical analysis are provided in Supplemental Table S1. Amino acids were categorized after their respective common precursor. PEP: phosphoenolpyruvate. D, Analysis of the breakdown products of Leu, Ile, and Val—KIC, KIV, and KMV—and phenylpyruvate (PhePyr) in seedlings of WT compared to *GRXS15 K83A* lines #3 and #4. WT was set to 100% (*n* = 4–5; means ± sem). Absolute values are provided in Supplemental Table S1. The statistical analysis (two-way ANOVA with post hoc Holm–Sidak comparisons for WT versus *grxs15* mutant) indicated significant changes; **P ≤ 0.01; ***P ≤ 0.001.

The pronounced pyruvate accumulation may be caused by a backlog of metabolites due to a lower TCA flux or by a diminished activity of PDC, which catalyzes the decarboxylation of pyruvate to acetyl-CoA (Yu et al., 2012). The E2 subunit of this multi-enzyme complex uses an LC, the synthesis of which was shown to be compromised in *GRXS15^amiR^* mutants (Ströher et al., 2016). In plant mitochondria, the lipoyl moiety is an essential cofactor of four protein complexes: PDC, OGDC, BCKDC, and GDC (Taylor et al., 2004). Ströher et al. (2016) showed decreased lipoylation of PDC E2-2 and E2-3 but no effects on E2-1. On the other hand, a pronounced decrease was observed in all GDC H protein isoforms, and differences in the degree of lipoylation were explained by different modes of lipoylation. To get insight into the metabolic effects of diminished GRXS15 activity, we tested for protein lipoylation in the weakest complementation line #4 and directly compared the results to lipoylation in *GRXS15^amiR^* and WT. GRXS15 was barely detectable in *GRXS15^amiR^* while in line #4 the mutated GRXS15 K83A had been shown earlier to be present at even higher amounts than the endogenous protein in WT plants (Moseler et al., 2015; Ströher et al., 2016). Immunodetection of the lipoyl group with specific antibodies to the cofactor indicated that the amount of lipoate bound to the H subunit isoforms of GDC was decreased in the *GRXS15 K83A* mutant to a similar extent as in *GRXS15^amiR^* (Figure 7A). In contrast, the H protein levels were largely unchanged in all tested lines. Effective lipoylation could be restricted by the activity or the abundance of mLIP1. An activity assay for mLIP1 in plant extracts has not been developed. Using antibodies raised against recombinant mLIP1, there was no difference in the protein levels between line #4, *GRXS15^amiR^*, and WT controls (Figure 7A). However, this does not exclude an enzymatic defect in mLIP1.

To further test whether the accumulation of pyruvate was due to a less active PDC, we measured the activity of the PDC in isolated mitochondria. Interestingly, there was a 22% reduction in PDC activity for the *GRXS15 K83A* mutant. While the WT had a PDC activity of 92.7 ± 6.5 nmol NADH mg^−1^ min^−1^ the *GRXS15 K83A* line #3 had significantly lower activity of only 72.40 ± 6.2 nmol NADH mg^−1^ min^−1^ (Figure 7B).

The pronounced increase of pyruvate and several TCA intermediates (Figure 6) may have further effects on downstream metabolites. Given that intermediates of glycolysis and the TCA cycle are hubs for synthesis of amino acids and because mutants defective in PDC subunit E2 show an increase in the pools of nearly all amino acids (Yu et al., 2012), we profiled the abundance of amino acids. Most amino acids were increased in the mutants compared to WT seedlings, with more pronounced increases in line #4 compared to line #3 (Figure 7C;Supplemental Table S1). Particularly, high increases in amino acid abundance of ˃ 200% were observed for glycine and serine derived from 3-phosphoglycerate (3PG), for alanine, Leu, and Val all derived from pyruvate, and for Ile (Figure 7C;Supplemental Table S1). The Gly/Ser ratio, indicative of photorespiratory effects, did not show any pronounced change and varied only between 0.33 ± 0.04 for the WT, 0.4 ± 0.1 for line #3, and 0.37 ± 0.12 for line #4.

### BCAA metabolism is strongly impaired in response to diminished GRXS15 activity and LC availability

Leu, Val, and Ile are classified as BCAAs, which share a common degradation pathway that is localized in the mitochondrion. Because the BCAA catabolism pathway involves LC-dependent BCKDC, the increase in the pools of all three BCAAs may not exclusively result from increased availability of their parent compounds, but also from restricted BCAA degradation capacity. To test this hypothesis, we measured the content of the respective keto acids resulting from deamination of the BCAAs by BCAA transaminase (BCAT; Supplemental Figure S6A). The keto acids α-ketoisocaproic acid (KIC), α-keto-β-methylvaleric acid (KMV), and α-ketoisovaleric acid (KIV) derived from the BCAAs accumulated massively in both *GRXS15 K83A* mutants (Figure 7D). Here, KIC accumulated in the *GRXS15 K83A* mutants up to 15-fold, resulting in values of 3.5 ± 0.11 pmol (mg FW)^−1^ in line #3 and 3.8 ± 0.6 pmol (mg FW)^−1^ in line #4 compared to 0.25 ± 0.032 pmol (mg FW)^−1^ in the WT. KIV and KMV increased six- to seven-fold in the *GRXS15 K83A* mutants. These pronounced changes support the hypothesis of decreased BCKDC activity creating a bottleneck in keto acid catabolism (Supplemental Figure S6A). The higher accumulation of KIC can be accounted for by the preference of BCKDC for the Val derivative (Taylor et al., 2004) resulting in KIV to be metabolized faster and to accumulate less strongly. Despite the presumed bottleneck in catabolism of BCAAs, the *grxs15* mutants did not show enhanced Leu sensitivity (Supplemental Figure S6B). Similarly, *ivdh* mutants deficient in isovaleryl-CoA dehydrogenase did not display an increased sensitivity to external supply of Leu compared to WT. To exclude that the accumulation of keto acids is just due to an increased abundance of the respective BCAAs, we measured the BCAAs and their deaminated keto acids in the mutant *root meristemless1* (*rml1*; Cheng et al., 1995; Vernoux et al., 2000). *rml1* is severely compromised in glutathione biosynthesis, and is characterized by only residual amounts of glutathione, accumulation of cysteine, and largely inhibited growth (Vernoux et al., 2000). We found a significant increase in all three BCAAs in *rml1* compared to the WT but no accumulation of the respective keto acids (Supplemental Figure S7). Hence, the accumulation of BCAAs alone cannot account for an increase of KIV, KIV, and KMV.

## Discussion

### GRXS15 function limits growth

Null mutants of mitochondrial GRXS15 are embryo-defective but can be partially complemented with a mutated GRXS15 protein compromised in its ability to coordinate a [2Fe–2S] cluster (Moseler et al., 2015). The bottleneck in Fe–S coordination results in a dwarf phenotype similar to the phenotype of severe knockdown mutants generated through expression of artificial microRNAs (Supplemental Figure S1; Ströher et al., 2016) but how exactly the modification of either activity or abundance of GRXS15 impacts on plant growth and development remained unclear. Less severe knockdown mutants resulting from a T-DNA insertion in the 5′-UTR of *GRXS15* limited the abundance of GRXS15 to ∼20% of WT levels, but did not show a macroscopic phenotype beyond early seedling stage under nonstress conditions (Ströher et al., 2016). The growth phenotype of more severe *grxs15* mutants is most apparent in very short roots, which may be linked to the fact that *GRXS15* is strongly expressed in roots, particularly in the maturation and meristematic zone (Belin et al., 2015). The primary function of GRXS15 is assumed to be a role in mitochondrial Fe–S cluster transfer (Moseler et al., 2015; Ströher et al., 2016). Recently, an interaction of GRXS15 with ISCA scaffold proteins and a transfer of a [2Fe–2S] cluster from GRXS15 to the ISCA complex was shown in vitro (Azam et al., 2020). This implies that a compromised GRXS15 function potentially may have implications for Fe–S-dependent pathways, including biosynthesis of biotin and Moco, the mETC, and the TCA cycle. While biotin feeding experiments clearly excluded biotin biosynthesis as the limiting factor, the picture was less clear for Moco, which is an essential cofactor for several cytosolic enzymes (Schwarz and Mendel, 2006). Nitrate assimilation, which is dependent on Moco-containing NR, initially showed the expected nitrate sensitivity. Measurements of extractable NR activity, however, showed no defects. Because, similarly XDH and AOs did not show changes in their activities between mutants and the WT, deficiencies in Moco supply can be excluded as a putative metabolic bottleneck in *GRXS15 K83A* mutants. Nitrate sensitivity in *grxs15* mutants leaves us with the conundrum of a different link between mitochondrial functions of GRXS15 and nutrient assimilation, which deserves further investigation in the future.

### GRXS15 does not affect energy balance and ROS levels

Diminished growth coincides with decreased root respiration rates in both, severe *GRXS15^amiR^* knockdown mutants (Ströher et al., 2016) and the weak complementation line #3 investigated in this work (Figure 4A). Because the mETC contains 12 Fe–S proteins involved in electron transport (Couturier et al., 2015; Meyer et al., 2019), restricted supply of Fe–S clusters would be expected to affect electron flow along the mETC. In humans (*Homo sapiens*), it was observed that a patient deficient in mitochondrial GLRX5 suffers from decreased abundance and hence activity of complex I (Ye et al., 2010). In yeast, *Δgrx5* mutants displayed a decreased complex II activity, albeit an unaffected protein abundance in this complex (Rodríguez-Manzaneque et al., 2002). In contrast, we found no changes in abundance of any mETC complexes in severe *grxs15* mutants of Arabidopsis (Figure 4B). Consistently, feeding of mitochondria isolated from *GRXS15 K83A* mutants with succinate revealed that SDH, which contains three different Fe–S clusters (Figueroa et al., 2001), does not constitute a bottleneck in mitochondrial metabolism of *grxs15* mutants. Generally, the respiratory capacity is not affected in the mutants compared to WT, which indicates that supply of Fe–S clusters to components of the mETC is not compromised in *grxs15* mutants. The lower respiratory rate in *GRXS15 K83A* mutants also does not lead to changes in ATP levels. This, however, may also partially be due to decreased consumption of ATP with restricted growth and also the activity of adenylate kinase that contributes to formation of ATP (and AMP) from ADP to buffer the ATP level (De Col et al., 2017). Our overall conclusion to this point is that reduced respiration is likely due to restricted substrate supply rather than assembly of complexes in the mETC and their supply with Fe–S clusters. Restricted supply of reducing equivalents may result from a slowdown of the TCA cycle and also from severely compromised contributions of the electron-transfer flavoprotein (ETF)/ETF:ubiquinone oxidoreductase (ETF/ETFQO) to ubiquinone reduction (Supplemental Figure S6). Electrons that enter the mETC via ETF/ETFQO originate from IVDH-mediated oxidation of acyl-CoAs as products of BCKDC. The ETF/ETFQO pathway has been shown to contribute significant amounts of electrons in stress situations (Ishizaki et al., 2005; Pires et al., 2016). The concomitant increase in BCKAs and particularly BCAAs may contribute to the dwarf phenotype as disruption in BCAA homeostasis has been shown to lead to pleiotropic effects including growth retardation (Cao et al., 2019).

### GRXS15 affects enzymes and metabolites in the TCA cycle

GRXS15 was detected as part of higher-order protein assemblies in a mitochondrial complexome analysis (Senkler et al., 2017). A particularly strong interaction between GRXS15 and mitochondrial IDH1 was observed in yeast two-hybrid screens with IDH1 as bait and this interaction was subsequently confirmed by bimolecular fluorescence assays (Zhang et al., 2018). Consistent with a suspected role of GRXS15 in IDH1 function, the isocitrate content was decreased significantly in a *grxs15* knockdown mutant, while the relative flux through the TCA cycle increased (Zhang et al., 2018). IDH1 has recently been reported to contain several redox-active thiols that can change their redox state depending on substrate availability for the TCA (Nietzel et al., 2020). The IDH1–GRXS15 interaction thus could point at a possible function of GRXS15 as a thiol-switch operator for regulatory thiols. This is unlikely though, because GRXS15 does not show any reductive activity and only weak oxidative activity (Moseler et al., 2015; Begas et al., 2017). The increase in all analyzed metabolites of the TCA cycle is rather consistent with metabolite patterns found in knockdown mutants of mitochondrial MnSOD, in which increased levels of organic acids correlated with a decrease in ACO activity (Morgan et al., 2008). ACO contains a [4Fe–4S] cluster and has frequently been used as an enzymatic marker for defects in Fe–S cluster assembly and transfer in yeast and human cells (Rodríguez-Manzaneque et al., 2002; Bandyopadhyay et al., 2008; Liu et al., 2016). It came as a surprise that ACO was reported to be unaffected in mitochondria of Arabidopsis *grxs15* mutants, both in abundance and activity (Ströher et al., 2016). Consistent with this report, we also found no change in abundance or activity of total ACOs and only a minor change in mitochondrial ACO activity (Figure 5). Thus, the unchanged ACO activity in *GRXS15 K83A* mutants does not explain the most pronounced increase in pyruvate, which accumulates up to five-fold and thus supersedes the accumulation of all other TCA cycle intermediates at least by a factor of two. A knockdown of mitochondrial and cytosolic ACO activities in wild tomato led to a reduction in 2-OG levels but an increase in citrate and isocitrate by 40%–50%. A similar change in these organic acids of the TCA cycle was found in an SDH mutant (Carrari et al., 2003; Huang et al., 2013). The pattern of organic acids in *GRXS15 K83A* mutants is thus clearly different from other TCA cycle mutants.

### GRXS15 has a function in protein lipoylation

PDC and OGDC do not contain an Fe–S cluster but rather belong to a class of four dehydrogenase complexes that all involve lipoylated subunits. Decreased lipoylation of GDC-H proteins and reduced PDC activity is fully consistent with previous observations on *GRXS15^amiR^* mutants by Ströher et al. (2016). Additionally, the lack of changes in abundance of 2-OG found for *GRXS15 K83A* mutants in this work is consistent with the absence of detectable changes in lipoylation of OGDC-E2 reported by Ströher et al. Similar to the Arabidopsis mutants, humans carrying mutations in mitochondrial GLRX5 are also deficient in lipoylation of mitochondrial proteins (Baker et al., 2014). A deficiency in lipoylation in Arabidopsis *grxs15* mutants is further supported by increased amounts of pyruvate as well as several other organic acids and amino acids derived from pyruvate and 2-OG (Figures 6 and 7C). Similar increases in pyruvate, as well as the accumulation of most amino acids, were also shown for Arabidopsis plants with a mutated PDC-E2 subunit resulting in 30% PDC activity (Yu et al., 2012). A much more pronounced increase of alanine in *PDC-E2* mutants than in *GRXS15 K83A* mutants may be attributed to a higher severity of the metabolic bottleneck if PDC activity is down to 30%. Of all metabolites analyzed in this study, the 4- to 15-fold increases of BCKAs in *GRXS15 K83A* mutants were the most pronounced relative changes compared to the WT. The findings that these increases were stronger in more severe mutants, point at BCKDC as a critical bottleneck. The keto acids, KIC, KIV, and KMV are products of transamination of the BCAAs Leu, Ile, and Val (Hildebrandt et al., 2015). Further degradation of the keto acids in *GRXS15 K83A* mutants is limited because BCKDC relies on efficient lipoylation of the E2 subunit. Indeed, selective accumulation of BCAAs, but not of the respective keto acids, in the *rml1* mutant supports the notion that the increase in BCKAs in GRXS15 K83A is likely due to metabolic restriction in further degradation of the keto acids.

**Figure 8:**
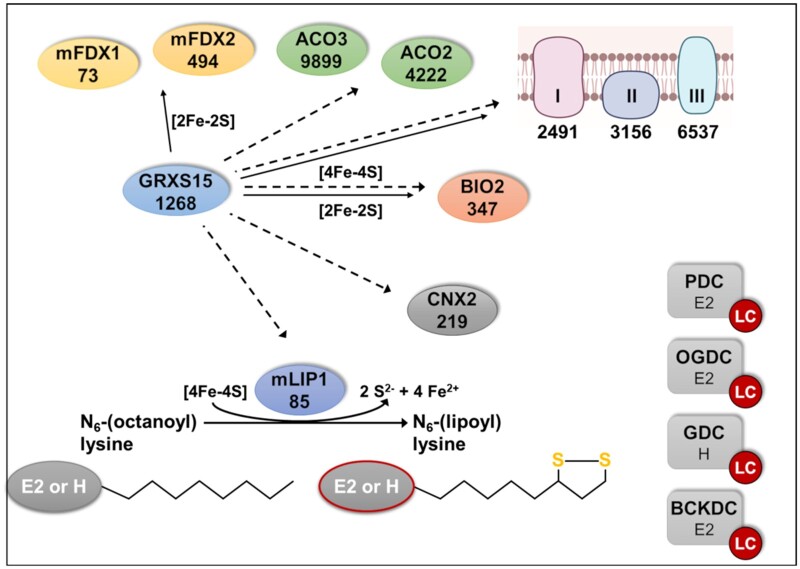
Lipoylation of mitochondrial proteins depends on GRXS15. Distribution of Fe–S clusters in Arabidopsis mitochondria to Fe–S proteins and lipoylation of proteins via mLIP1. Putative transfer of Fe–S clusters is indicated by solid arrows for [2Fe–2S] and dashed arrows for [4Fe–4S]. Intermediate complexes of Fe–S transfer and assembly of [4Fe–4S] clusters are not shown. mFDX1/2: mitochondrial ferredoxin 1/2; ACO2/3: aconitase 2/3; BIO2: biotin synthase 2; I, II, and III represent complexes I (2 [2Fe–2S], 6 [4Fe–4S]), II (1 [2Fe–2S], 1 [3Fe–4S], 1 [4Fe–4S]), and III (1 [2Fe–2S]) of the mETC; E2 and H are the lipoylated subunits of the four dehydrogenase complexes PDC, GDC, OGDC, and BCKDC. Numbers indicate the estimated copy number of the respective proteins or protein complexes (copy number of Fe–S coordinating subunits in the mETC are listed in Supplemental Table S2) according to Fuchs et al. (2020) which is based on mito from a heterotrophic Arabidopsis cell culture.

In summary, decreased activity of the mitochondrial GRXS15 appears to selectively restrict protein lipoylation. Lipoylation of mitochondrial proteins is mediated through coordinated action of lipoate-protein ligase, octanoyltransferase, and mLIP1 (Ewald et al., 2014). mLIP1 contains two [4Fe–4S] clusters, which link the function of this enzyme to the Fe–S cluster transfer machinery (Balk and Schaedler, 2014). At a first glance, a selective defect in only one out of about 26 mitochondrial [4Fe–4S] proteins (Przybyla-Toscano et al., 2021) may seem surprising and poses the question for an explanation. Either mLIP1 has a specific requirement for GRXS15 in the assembly of its Fe–S cofactors, or mLIP1 is more sensitive than other Fe–S enzymes to restricted Fe–S cluster supply. However, none of these hypotheses are as yet supported by available experimental data. A third possible explanation is based on established protein abundance. Recently, Fuchs et al. (2020) reported quantitative data for the abundance of proteins in single mitochondria (Figure 8). mLIP1 was estimated to be present with only 85 copies in a single mitochondrion compared to 4,200 copies of ACO2 and 9,900 copies of ACO3 (Figure 8; Fuchs et al., 2020). In the absence of any other evidence, we have to assume that all apoproteins have a similar likelihood of receiving a [4Fe–4S] cluster based on random interactions of transfer proteins with apoproteins. In case of a compromised Fe–S supply, there will be an equal decrease of supply to all recipients but, in relative terms, mLIP1 with 85 copies might be stronger compromised than other recipients with higher copy numbers. This hypothesis needs to be tested in future work.

At this point an alternative mechanistic explanation for the selective effect of GRXS15 deficiency on protein lipoylation appears most plausible. The radical *S*-adenosylmethionine enzyme mLIP1 contains two [4Fe–4S] clusters one of which is required as a substrate, that is, as sulfur donor to octanoyl-residues (Figure 8; McCarthy and Booker, 2017). Continuous destruction of Fe–S clusters during lipoylation may thus render LC-dependent enzymes indirectly sensitive to defects in Fe–S supply. With the need for one [4Fe–4S] cluster to be replaced after each catalytic cycle and thus, having a higher turnover rate, the bottleneck is bound to become even more severe than in enzymes that use their Fe–S clusters only for electron transfer reactions. The notion that supply of LC is close to demand, has been observed in a study overexpressing H-protein, which negatively affected lipoate supply to E2 subunits of PDC and OGDC in the roots (López-Calcagno et al., 2019).

## Conclusion

We show that compromising the ability of GRXS15 to coordinate [2Fe–2S] clusters results in severe defects only in enzymes relying on LC. These results are in agreement with findings by Ströher et al. (2016) who reported diminished lipoylation of proteins in *GRXS15^amiR^* lines and hypothesized that diminished respiration and the short root mutant phenotype could be a consequence of the incomplete LC loading of important TCA cycle enzymes. Here we expand and specify the picture, by systematically probing for metabolic bottlenecks in mitochondrial pathways that rely on supply with Fe–S clusters. While changes in several metabolites were found, the primary defects can be assigned to the mitochondrial dehydrogenase complexes, all of which contain a lipoylated subunit. Those results emphasize the importance of mLIP1 as an important sink for Fe–S clusters, which becomes manifest if GRXS15-mediated Fe–S cluster transfer is restricted. The fact that most other Fe–S-dependent pathways are not seriously affected by deficiencies in *GRXS15 K83A* complementation lines may be explained by the effective relative abundance of different proteins in mitochondria. We propose that an increased demand for Fe–S as sulfur donor combined with the very low abundance of mLIP1 leads to the manifestation of a potentially lethal bottleneck.

## Material and methods

### Plant material and methods

Previously described Arabidopsis (*A. thaliana)* complementation lines *grxs15-3 UBQ10_pro_:GRXS15 K83A* (Moseler et al., 2015) and the knockdown line *GRXS15^amiR^* (Ströher et al., 2016), as well as *atm3-1* and *atm3-4* (Teschner et al., 2010) were used in this study. Arabidopsis ecotype Col-0 ([L.] Heyn.) segregated from the T-DNA line *grxs15-3*) was used as WT. Unless stated differently, surface-sterilized seeds were grown on vertical plates containing nutrient medium (Meyer and Fricker, 2000) with 0.1% (w/v) sucrose solidified with 0.8% (w/v) agar under long-day conditions with a diurnal cycle of 16-h light at 22°C and 8-h dark at 18°C. The light intensity was 75 μE m^−2^ s^−1^ and 50% air humidity.

Germination rate was scored by observing radical emergence in seeds plated on vertical culture plates using a stereomicroscope (M165 FC; Leica, Wetzlar, Germany ). Root growth was documented photographically on vertical culture plates containing 0.8% (w/v) phytagel and 0.1% (w/v) sucrose. Five and 8 d after stratification, root length was documented and measured using Adobe Illustrator CS5.1.

Influence of the nitrogen source on root length was analyzed on plates containing 5 mM KNO_3_ or 2.5 mM (NH_4_)_2_SO_4_, 2.5 mM KH_2_PO_4_, 2 mM MgSO_4_, 2 mM CaCl_2_, 50 μM Fe-EDTA, 70 µM H_3_BO_4_, 14 µM MnCl_2_, 0.5 µM CuSO_4_, 1 µM ZnSO_4_, 0.2 µM NaMoO_4_, 10 µM NaCl, 0.01 µM CoCl_2_, 0.8% (w/v) phytagel, and 0.1% (w/v) sucrose, pH 5.8. To check for possible effects of counter anions, (NH_4_)_2_SO_4_ was replaced by NH_4_Cl and grown otherwise exactly under the same conditions.

### Blue Native–PAGE

Mitochondrial samples were solubilized in 1% (w/v) *n*-dodecyl β-d-maltoside and subjected to Blue Native–PAGE as described previously (Meyer et al., 2011; Kühn et al., 2015).

### Isolation of mitochondria

Arabidopsis mitochondria were purified from 2- or 4-week-old seedlings as described before (Sweetlove et al., 2007) with slight modifications. All steps were performed on ice or at 4°C. Seedlings were homogenized using mortar and pestle and the homogenate was filtered (Miracloth; Merck Millipore, Burlington, MA, USA) before cellular debris was pelleted by centrifugation for 5 min at 1,200 *g*. The supernatant was centrifuged for 20 min at 18,000 *g*, and the pellet of crude mitochondria was gently resuspended in wash buffer (0.3 M sucrose, 0.1% (w/v) BSA and 10 mM TES, pH 7.5) and centrifuged for 5 min at 1,200 *g*. The supernatant was transferred into a new tube and centrifuged for 20 min at 18,000 *g*. The pellet was gently resuspended in final wash buffer (0.3 M sucrose, 10 mM TES, pH 7.5), loaded directly on a 0–6% (v/v) Percoll gradient and centrifuged for 40 min at 40,000 *g*. Mitochondria were transferred into a new tube and washed 3 times with final wash buffer (0.3 M sucrose, 10 mM TES pH 7.5).

### Respiration assays

O_2_ consumption of intact Arabidopsis roots and isolated mitochondria was measured in Oxytherm Clark-type electrodes (Hansatech, Norfolk, UK; www.hansatech-instruments.com) as described before (Wagner et al., 2015). Whole roots from seedlings vertically grown on agar plates were cut below the hypocotyl-root junction and assayed in a volume of 1.2 mL containing 5 mM KCl, 10 mM MES, and 10 mM CaCl_2_, pH 5.8, and after addition of 4 mM KCN and 0.2 mM pGal.

O_2_ consumption of isolated mitochondria was measured in a volume of 1 mL containing 0.3 M mannitol, 10 mM TES-KOH pH 7.5, 5 mM KH_2_PO_4_, 10 mM NaCl, 2 mM MgSO_4_, and 0.1% (w/v) bovine serum albumin. O_2_ consumption rate was measured before (blank) addition of mitochondria and after addition of mitochondria or respective substrate; State II; succinate (10 mM succinate, 0.25 mM ATP) or pyruvate/malate (10 mM pyruvate, 10 mM malate, 0.3 mM NAD and 0.1 mM thiamine pyrophosphate), State III; ADP (50 μM ADP). Additionally, O_2_ consumption rate was analyzed after ADP consumption (State IV) and after addition of 10 μM carbonyl cyanide m-chlorophenylhydrazone (CCCP).

### Histological detection of ROS

For detection of increased H_2_O_2_ production, leaves were stained with DAB (Thordal-Christensen et al., 1997). Leaves were vacuum-infiltrated in a solution containing 0.1 mg mL^−1^ DAB, 50 mM potassium phosphate buffer pH 7.6 and 0.1% (v/v) Silwet L-77. After infiltration, the leaves were incubated for 20–24 h in the dark and destained by lactic acid:glycerol:EtOH (1:1:3) for 30 min at 70°C.

For histochemical staining of superoxide, NBT was used (Hoffmann et al., 2013). Leaves were vacuum-infiltrated in a solution containing 0.1 mg mL^−1^ NBT, 50 mM potassium phosphate buffer pH 7.6 and 0.1% (v/v) Silwet L-77. After infiltration, the leaves were incubated for 30 min in the dark and destained by lactic acid:glycerol:EtOH (1:1:3) for 30 min at 70°C.

### Determination of metabolite levels via HPLC

Aliquots (45–55 mg) of freshly ground plant tissue were used for absolute quantification of amino acid, α-keto acid, and organic acid content each.

Free amino acids and α-keto acids were extracted with 0.5 mL ice-cold 0.1 M HCl in an ultrasonic ice bath for 10 min. Cell debris and insoluble material were removed by centrifugation for 10 min at 25,000 *g*. For the determination of α-keto acids, 150 µL of the resulting supernatant were mixed with an equal volume of 25 mM o-phenylendiamine solution and derivatized by incubation at 50°C for 30 min. After centrifugation for 10 min, the derivatized keto acids were separated by reversed phase chromatography on an Acquity HSS T3 column (100 mm × 2.1 mm, 1.7 µm; Waters, Milford, USA) connected to an Acquity H-class UPLC system. Prior separation, the column was heated to 40°C and equilibrated with five column volumes of solvent A (0.1% [v/v] formic acid in 10% [v/v] acetonitrile) at a flow rate of 0.55 mL min^−1^. Separation of keto acid derivatives was achieved by increasing the concentration of solvent B (acetonitrile) in solvent A (2 min 2% (v/v) B, 5 min 18% (v/v) B, 5.2 min 22% (v/v) B, 9 min 40% (v/v) B, 9.1 min 80% (v/v) B and hold for 2 min, and return to 2% (v/v) B in 2 min). The separated derivatives were detected by fluorescence (Acquity FLR detector, Waters, excitation: 350 nm, emission: 410 nm) and quantified using ultrapure standards (Sigma, St Louis MO, USA). Data acquisition and processing were performed with the Empower3 software suite (Waters). Derivatization and separation of amino acids were performed as described by Yang et al. (2015).

Total organic acids were extracted with 0.5 mL ultra-pure water for 20 min at 95°C. Organic acids were separated using an IonPac AS11-HC (2 mm; Thermo Scientific, Waltham, MA, USA) column connected to an ICS-5000 system (Thermo Scientific) and quantified by conductivity detection after cation suppression (ASRS-300 2 mm, suppressor current 95–120 mA). Prior separation, the column was heated to 30°C and equilibrated with five column volumes of solvent A (ultra-pure water) at a flow rate of 0.38 mL min^−1^. Separation of anions and organic acids was achieved by increasing the concentration of solvent B (100 mM NaOH) in buffer A (8 min 4% (v/v) B, 18 min 18% (v/v) B, 25 min 19% (v/v) B, 43 min 30% (v/v) B, 53 min 62% (v/v) B, 53.1 min 80% (v/v) B for 6 min, and return to 4% (v/v) B in 11 min). Soluble sugars were separated on a CarboPac PA1 column (Thermo Scientific) connected to the ICS-5000 system and quantified by pulsed amperometric detection . Column temperature was kept constant at 25°C and equilibrated with five column volumes of solvent A (ultra-pure water) at a flow rate of 1 mL min^−1^. Baseline separation of carbohydrates was achieved by increasing the concentration of solvent B (300 mM NaOH) in solvent A (from 0 to 25 min 7.4% (v/v) B, followed by a gradient to 100% (v/v) B within 12 min, hold for 8 min at 100% (v/v) B, return to 7.4% (v/v) B and equilibration of the column for 12 min). Data acquisition and quantification were performed with Chromeleon 7 (Thermo Scientific).

### AO and XDH assay

AO and XDH assays were performed similar as described previously by Koshiba et al. (1996) and Hesberg et al. (2004). For determination of AO and XDH activities, Arabidopsis seedlings were homogenized in extraction buffer (0.1 M potassium phosphate buffer pH 7.5, 2.5 mM EDTA and 5 mM DTT) and centrifuged for 10 min at 16,000 *g* and 4°C. Enzyme activities of AO and XDH in the resulting supernatant were detected after native PAGE by activity staining. Activity of AO was developed in a reaction mixture containing 0.1 M potassium phosphate buffer pH 7.5, 1 mM 1-naphthaldehyde, 1 mM indole-3-carboxaldehyde, 0.1 mM phenazine methosulfate (PMS), and 0.4 mM 3-(4,5-dimethylthiazol-2-yl)-2,5-diphenyltetrazolium bromide (MTT). Activity of XDH was analyzed with a staining solution of 1 mM hypoxanthine, 1 mM MTT, and 0.1 mM PMS in 250 mM Tris–HCl, pH 8.5.

### NR assay

NR assay was performed as described previously (Scheible et al., 1997) with slight modifications. Leaves were homogenized in extraction buffer (50 mM 3-(N-morpholino)propanesulfonic acid (MOPS), pH 7.0, 50 mM KCl, 5 mM Mg acetate, 1 mM CaCl_2_, 2 mM Na-citrate, and 1 mM DTT) and centrifuged for 10 min at 20,000 *g* and 4°C. NR activity was measured in a reaction mixture containing 50 mM MOPS, pH 7.0, 50 mM KCl, 5 mM Mg acetate, 1 mM CaCl_2_, 10 mM KNO_3_, and 0.4 mM NADH. At consecutive time points, 150 µL aliquots were removed from the mixture and the reaction was stopped by adding 54 mM zinc acetate and 37.5 µM PMS. Thereafter, 0.475% (v/v) sulfanilamide in 1 N HCl and 0.005% (v/v) *N*-(1-naphthyl)-ethylenediamine was added. Samples were allowed to stand for 15 min at RT in the dark and the absorbance of the produced azo-dye was measured at 540 nm.

### ACO assay

ACO activity in cell extracts and purified mitochondria was assayed by coupling this activity to IDH and measuring the formation of NADPH. Leaf tissue was homogenized in 1.5 volumes of 50 mM Tris–HCl pH 8, 50 mM KCl, 0.2% (v/v) Triton X-100, 2 mM sodium citrate and 1 mM dithiothreitol, followed by centrifugation at 13,000*g* for 10 min at 4°C to remove cell debris. Each sample was processed immediately before activity measurement, except where stated, to avoid inactivation by O_2_. The reaction (1 mL) contained 100–150 µg of leaf protein extract or 15–20 μg mitochondrial protein in 0.1 M Tris–HCl pH 8, 1.5 mM MgCl_2_, 0.1% (v/v) Triton X-100, 1 mM NADP^+^ and 0.36 U IDH. After 20–30 s of equilibration, the reaction was started by adding 0.15 mM cis-aconitic acid. The formation of NADPH was followed over time by measuring the increase in absorbance at 340 nm in a JASCO V-550 spectrophotometer.

### PDC assay

To estimate the activity of PDC, mitochondria were isolated as described previously and reduction of NAD^+^ was measured at 340 nm in a reaction mixture containing ∼10 µg mitochondria in 100 mM MOPS pH 7.4, 1 mM CaCl_2_, 1 mM MgCl_2_, 4 mM cysteine, 0.45 mM thiamine pyrophosphate, 0.18 mM Coenzyme A, 3 mM NAD^+^ and 0.1% (v/v) Triton X-100. The reaction was started with 7.5 mM pyruvate.

### Fatty acid methyl ester measurement

The analysis of fatty acids was performed by quantification of their respective fatty acid methyl esters (FAMEs) via gas chromatography coupled with a flame ionization detector as described before (Browse et al., 1986). One milliliter 1 N HCl in methanol was added to five seeds or ∼50 mg homogenized seedlings as well as 5 µg pentadecanoic acid as internal standard. Samples were incubated at 80°C for 2 h (seeds) or 30 min (seedlings). After cooling down, 1 mL 0.9% (w/v) NaCl and 1 mL hexane were added. Samples were mixed vigorously and centrifuged with 1,000 *g* for 3 min. The hexane phase was transferred to a GC vial. FAMEs were quantified using pentadecanoic acid as internal standard.

### Western blotting

For protein blot analysis, total cell extract or purified organelles were heated for 5 min and separated on standard sodium dodecyl sulfate-PAGE. Proteins were transferred to a membrane (BioTrace PVDF Transfer Membrane; Pall Corporation, Port Washington, NY, USA) and labeled with antibodies (Streptavidin HRP: ab7403; Abcam, Cambridge, UK; lipoic acid: ab58724, ACO): see Bernard et al. (2009). Antibodies against GRXS15 and mLIP1 were kindly provided by Nicolas Rouhier and Jonathan Przybyla-Toscano (Nancy) and the H protein antibody a kind gift of Olivier Keech (Umea). The TOM40 antibody was a kind gift of Jim Whelan (Melbourne). Immunolabeling was detected by chemiluminescence by using secondary horseradish peroxidase-conjugated antibodies and Pierce ECL Western Blotting Substrate.

### Fluorescence microscopy

Fluorescent plants were selected using a stereomicroscope (Leica M165 FC) equipped with a GFP filter.

A confocal laser scanning microscope (Zeiss LSM 780, attached to an Axio Observer.Z1; Carl Zeiss Microscopy) equipped with a solid state 405 nm laser and an Argon laser for excitation at 458 nm and 488 nm, and a ×40 (C-Apochromat, 1.20 numerical aperture, water immersion) or ×63 lens (Plan-Apochromat, 1.40 numerical aperture, oil immersion) was used for confocal imaging. For ratiometric analyses of mitochondrially localized roGFP2-hGrx1 (Albrecht et al., 2014) or roGFP2-Orp1 (Nietzel et al., 2019), lines with similar expression levels in WT and mutants were selected. For both sensors, roGFP2 was excited at 405 and 488 nm. For both excitation wavelengths, roGFP2 fluorescence was collected with a bandpass filter of 505–530 nm.

The cytosolic ATeam 1.03-nD/nA was excited at 458 nm and emission of CFP (mseCFP) and Venus (cp173-mVenus) was collected at 499–544 nm and 579–615 nm, respectively. Background signal was subtracted before ratiometric analysis.

For all emissions, intensities from four scans were averaged. Ratiometric analysis was performed using a custom-written MATLAB script (Fricker, 2016) using x,y noise filtering and fluorescence background subtraction.

### Statistical analysis

Statistics and error bars were applied for independent experiments with at least three biological replicates using the program GraphPad Prism version 6.

### Accession numbers

GRXS15 (At3g15660), BIO2 (At2g43360), MCCA (At1g03090), ATM3 (At5g58270), CNX2 (At2g31955), CNX3 (At1g01290), NR1 (At1g77760), NR2 (At1g37130), AAO1 (At5g20960), AAO2 (At3g43600), AAO3 (At2g27150), XDH1 (At4g34890) XDH2 (At4g34900), ACO1 (At4g35830), ACO2 (At4g26970), ACO3 (At2g05710), mLIP1 (At2g20860), IVDH (At3g45300), and BCAT (At5g65780).

## Supplemental data

The following materials are available in the online version of this article.

**Supplemental Figure S1.** Arabidopsis mutants affected in GRXS15 function develop a dwarf phenotype.

**Supplemental Figure S2.** Moco enzymes and anions are not affected in Arabidopsis *GRXS15 K83A* mutants.

**Supplemental Figure S3.** In vivo monitoring of ATP levels in the cytosol of Arabidopsis *GRXS15 K83A* mutants.

**Supplemental Figure S4.** Activity and stability of ACO in mitochondria and leaf extracts.

**Supplemental Figure S5.** Analysis of the oxidation state of the Arabidopsis *grxs15* mutants.

**Supplemental Figure S6.** Catabolism of BCAAs in Arabidopsis seedlings.

**Supplemental Figure S7.** BCAAs and their respective keto acids in the *rml1* mutant.

**Supplemental Table S1.** Content of amino acids and keto acids of Arabidopsis WT and *GRXS15 K83A* lines #3 and #4.

**Supplemental Table S2.** Fe–S cluster containing subunits of complexes of the mETC with the estimated copy numbers in mitochondria isolated from heterotrophic Arabidopsis cell culture.

## Supplementary Material

kiab172_Supplementary_Data

## Notes

A.M. and A.J.M. conceived the research with specific input from M.S. and J.B.; A.M. A.J.M., and J.B. designed the experiments and interpreted the data. I.K., A.E.M., M.F., S.W., R.W., K.F.S., G.P., M.W., T.M.H., and L.P. contributed experimental data, structural information and analyzed the data. A.M. and A.J.M. wrote the manuscript with support from M.S., T.H., M.W. R.H., P.D., and J.B.; A.J.M. agrees to serve as the author responsible for contact and ensures communication.

The author responsible for distribution of materials integral to the findings presented in this article in accordance with the policy described in the Instructions for Authors (https://academic.oup.com/plphys/pages/general-instructions) is: Andreas J. Meyer (andreas.meyer@uni-bonn.de).
