# Supplementary material for: The function of glutaredoxin GRXS15 is required for lipoyl-dependent dehydrogenases in mitochondria
Source: Plant Physiol. 2021 Apr 15;186(3):1507–25. doi: 10.1093/plphys/kiab172 (PMC8260144; doi:10.1093/plphys/kiab172)
Supplement: kiab172_Supplementary_Data [file kiab172_supplementary_data.pdf]

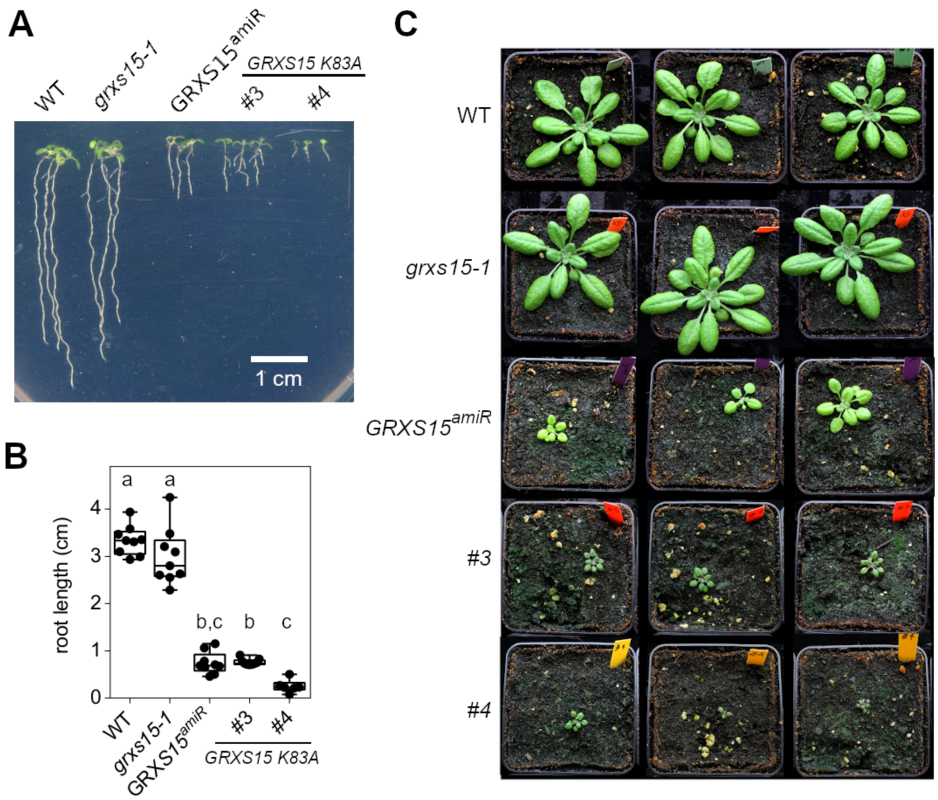

Supplemental Figure S1. Arabidopsis mutants affected in GRXS15 function develop a dwarf phenotype.

A, B: Growth of different *grxs15* mutants (*grxs15-1*, *GRXS15^{amiR}*, *GRXS15 K83A* lines #3 and #4) and wild-type (WT) seedlings on vertical plates with 0.8% agar under long-day conditions. Seedlings were documented and quantitatively analyzed for their root length 10 days after germination. (*n* = 6-9; the box plot shows the median as center line with the box for the first to the third quartile and whiskers indicating min and max values, points represent individual values of the whole data set). Different letters indicate significant differences between the different lines; $P \leq 0.05$; (one-way ANOVA).

C: Phenotypes of soil-grown plants after five weeks under long-day conditions (16 h light, 19°C, 8 h dark, 17°C; 50% rh).

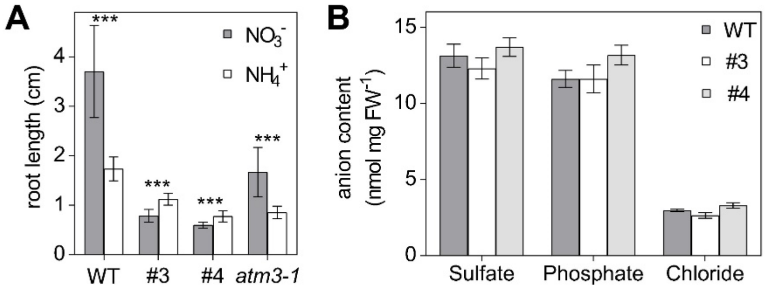

Supplemental Figure S2. Moco enzymes and anions are not affected in Arabidopsis *GRXS15 K83A* mutants

A: Primary root length of *GRXS15 K83A* lines #3 and #4 as well as *atm3-1* mutant seedlings compared to WT grown on vertical plates containing 5 mM KNO_3 or 5 mM NH_4Cl as N-source. Seedlings were grown for 9 days under long-day conditions (n = 35; means ± SD). Student's t-Test analysis showed significant differences between nitrate and ammonium treatment for each genotype (***: $P \leq 0.001$).

B: Amount of sulfate, phosphate and chloride in Arabidopsis WT and line #3 and #4 seedlings (n = 4; means ± SEM). The statistical analysis (two-way ANOVA with post hoc Holm-Sidak comparisons for WT vs. *grxs15*) indicated no significant ($P \leq 0.05$) change.

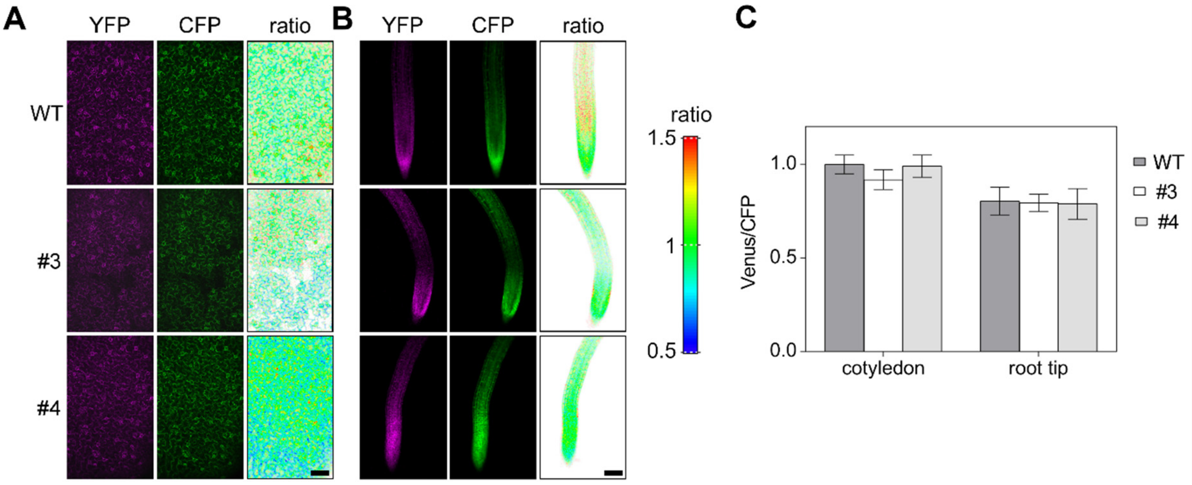

Supplemental Figure S3. ***In vivo*** **monitoring of ATP levels in the cytosol of Arabidopsis *GRXS15 K83A* mutants.**

A, B: ATeam1.03-nD/nA was stably expressed under a *35S* promoter in the cytosol of WT and *GRXS15 K83A* lines #3 and #4 and analyzed in cotyledon epidermis (A) and roots (B) for fluorescence intensities of Venus and CFP. Bars, 100 µm.

C: Venus/CFP fluorescence ratios calculated from fluorescence images of cotyledons and root tips of 7-d-old seedlings from two independent reporter lines for each genetic background (n = 10; means ± SD).

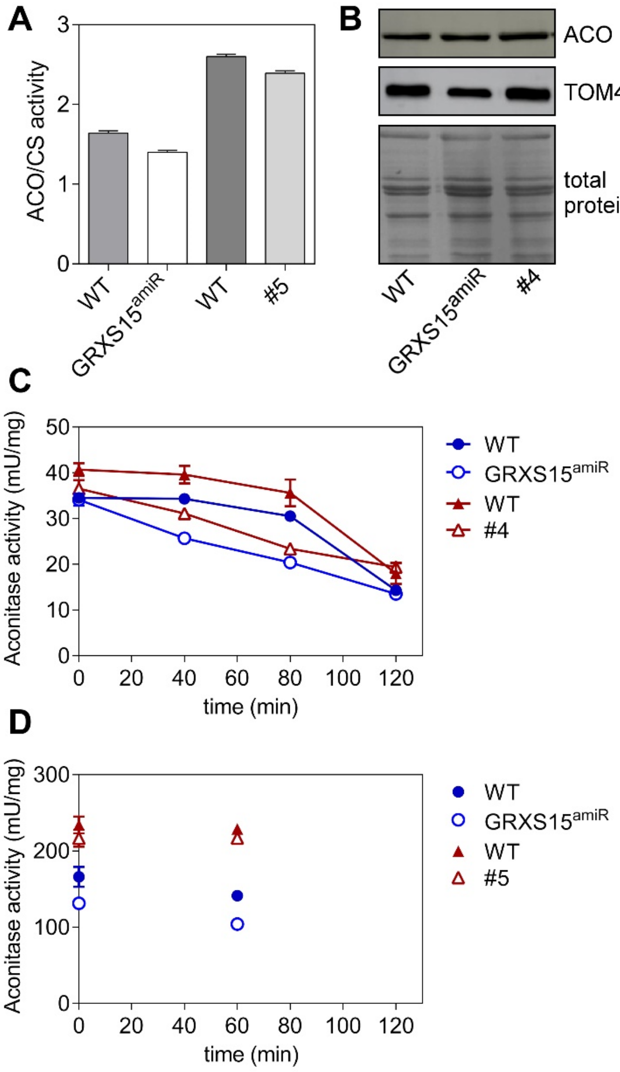

Supplemental Figure S4. Activity and stability of aconitase in mitochondria and leaf extracts.
A: Aconitase activity in isolated mitochondria, normalized by citrate synthase activity. On a protein basis, the values were 166; 131; 234 and 217 mU/mg protein for aconitase activity and 101, 94, 90 and 90 mU/mg protein for citrate synthase activity. Values are 2 technical replicates of pooled 4-week-old plants. Error bars represent the variability from the 2 measurements. Sibling line #5 of *GRXS15 K83A* was used for these assays, which is similar to line #4 in total leaf aconitase activity.
B: Protein gel blot analysis probed with antiserum raised against Arabidopsis ACO. 9 µg of mitochondrial protein of a wild-type plant as well as *GRXS15^{amiR}* and *GRXS15 K83A* lines #4 were loaded onto the gel. ACO and translocase of the mitochondria 40 (TOM40) protein levels were visualized by immunoblotting under denaturing conditions. Total protein staining served as a loading control.
C: Stability of aconitase in total leaf extracts. Each data point is the average of n = 2 leaf extracts with error bars indicating the difference divided by 2.
D: Stability of aconitase in isolated mitochondria. The data points are the average of 2 technical replicates from one biological sample (one mitoprep, from ~ 250 pooled seedlings). Error bars represent the variability from the 2 measurements.

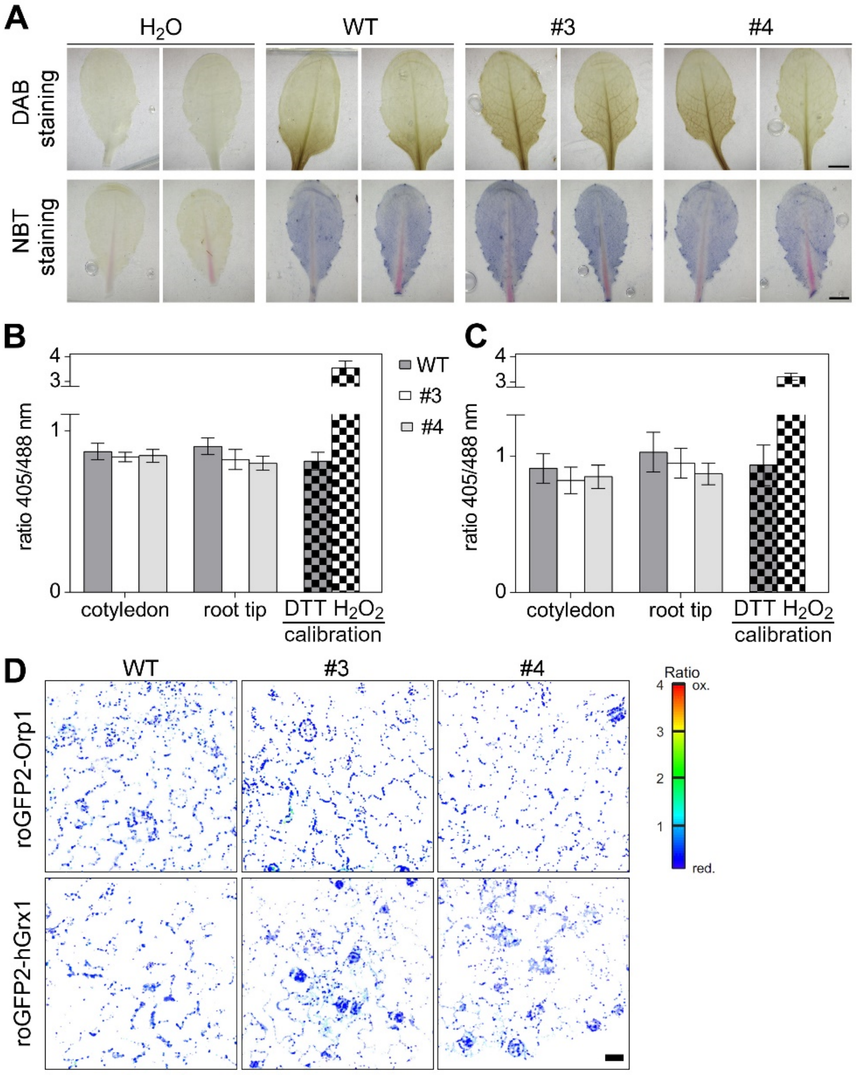

Supplemental Figure S5. Analysis of the oxidation state of the Arabidopsis *grxs15* mutants.

A: Representative images showing DAB (upper) and NBT (lower) staining for detection of increased ROS production in leaves. Wild-type plants and mutants were grown for four weeks under long-day growth conditions. Bars, 0.5 cm. *n* = 7-8.

B: Ratiometric analysis of the H_2O_2-sensitive fluorescent reporter roGFP2-Orp1. 7-d-old seedlings of WT and *GRXS15 K83A* lines #3 and #4 expressing mitochondrial roGFP2-Orp1 were analyzed for the redox state of the sensor in cotyledons and root tips. For estimation of the dynamic range of the sensor, wild-type seedlings were incubated in 10 mM DTT (grey squared) or 10 mM H_2O_2 (white squared) and fluorescence of roGFP2 in the hypocotyl was analyzed. Ratios were calculated from fluorescence images of cotyledons and root tips of 7-d-old seedlings from two independent reporter lines for each genetic background (*n* = 10; means ± SD).

C: Ratiometric analysis of the E_{GSH}–sensitive fluorescent reporter roGFP2-hGrx1 in mitochondria. Ratiometric analysis was performed with 7-d-old seedlings of WT and *GRXS15 K83A* lines #3 and #4 expressing mitochondrial roGFP2-hGrx1 by CLSM. For estimation of the dynamic range of the sensor, wild-type seedlings were incubated in 10 mM DTT (grey squared) or 10 mM H_2O_2 (white squared) and fluorescence of roGFP2 in the root tips was analyzed. Ratios were calculated from fluorescence images of cotyledons and root tips of 7-d-old seedlings from two independent reporter lines for each genetic background (*n* = 10; means ± SD).

D: Representative false color images of cotyledons of 7-d-old seedlings show the oxidation state of roGFP2-Orp1 or roGFP2-hGrx1 targeted to the mitochondrial matrix in WT and *GRXS15 K83A* lines #3 and #4. Bar, 20 µm.

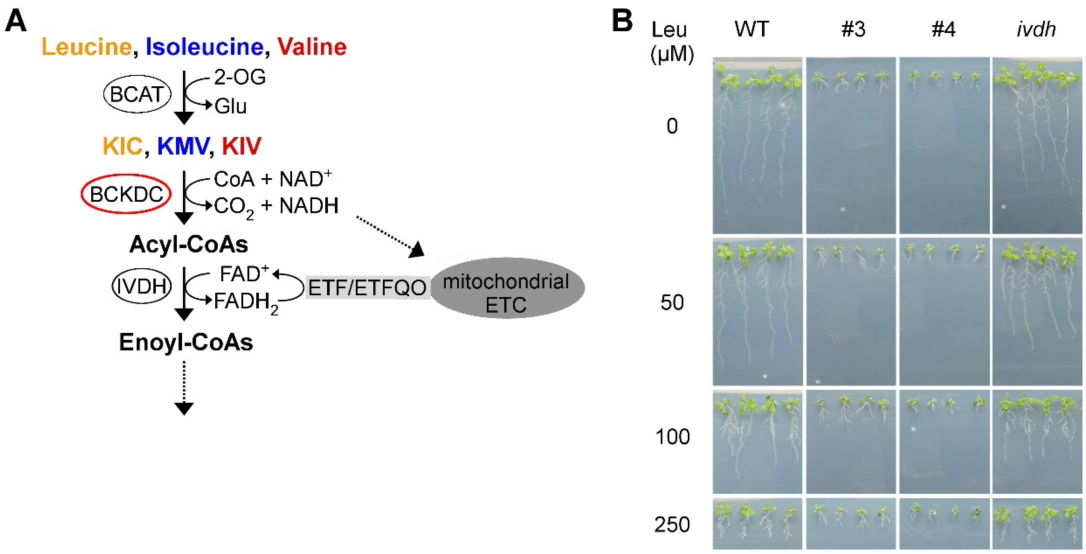

Supplemental Figure S6. Catabolism of branched-chain amino acids in Arabidopsis seedlings.
A: The branched-chain amino acids leucine, isoleucine and valine are deaminated by branched-chain aminotransferase (BCAT), which uses largely 2-oxoglutarate (2-OG) forming the branched-chain keto acids α-ketoisocaproic acid (KIC), α-keto-β-methylvaleric acid (KMV) and α-ketoisovaleric acid (KIV) as well as glutamate. The keto acids are further degraded by branched-chain keto acid dehydrogenase (BCKDC), which catalyzes the oxidative decarboxylation producing thereby acyl-CoA and NADH. Isovaleryl-CoA dehydrogenase (IVDH) catalyzes the third step providing electrons to the electron transport chain (ETC) via electron transfer flavoprotein (ETF)/electron transfer flavoprotein ubiquinone oxidoreductase (ETFQO) (modified after Peng *et al.*, (2015)).
B: Leucine sensitivity of WT, *GRXS15 K83A* lines #3 and #4 and *ivdh* mutants. 4-d-old seedlings were transferred to plates containing the respective leucine amount and were analyzed after 7 d.

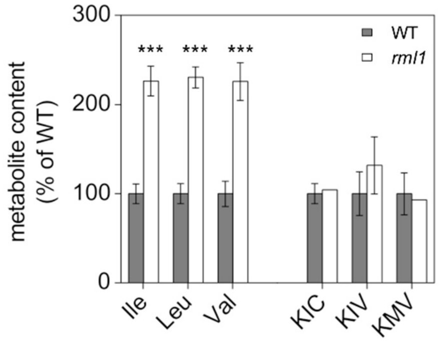

Supplemental Figure S7. Branched-chain amino acids and their respective keto acids in the *rml1* mutant.
Relative abundance of branched-chain amino acids Ile, Leu and Val and their degradation products, the branched-chain keto acids α-ketoisocaproic acid (KIC), α-keto-β-methylvaleric acid (KMV) and α-ketoisovaleric acid (KIV) in 8-d-old seedlings of *rml1* compared to WT seedlings. WT was set to 100% (n = 1-5, means ± SD). The statistical analysis (two-way ANOVA with post hoc Holm-Sidak comparisons for WT vs. *rml1* mutant) indicated significant changes; ***$P \leq 0.001$.

Supplemental Table S1. Content of amino acids and keto acids of Arabidopsis WT and G*RXS15 K83A* lines #3 and #4. The statistical analysis (two-way ANOVA with post hoc Holm-Sidak comparisons for WT vs. *grxs15* mutant) indicated significance levels; *n* = 4-5; *$P \leq 0.1$, **$P \leq 0.01$; ***$P \leq 0.001$; ns: not significant.

**Amino acid**	**amount of amino acid (pmol (mg FW)$^{-1}$); mean ± SEM (% compared to WT; significance level)**		
	**WT**	**#3**	**#4**
**Ala**	96.7 ± 6.2	206.5 ± 15.3 (213.6; ***)	238.0 ± 21.4 (246.2; ***)
**Leu**	6.5 ± 0.5	13.3 ± 1.4 (205.5; ***)	15.6 ± 1.3 (239.8; ***)
**Gly**	36.8 ± 4.8	67.7 ± 12.2 (184.0; ***)	82.3 ± 2.4 (223.6; ***)
**Ser**	111.7 ± 9.6	163.9 ± 9.1 (146.7; *)	234.6 ± 50.2 (209.9; ***)
**Val**	16.5 ± 0.7	25.5 ± 2.6 (154.5; *)	33.0 ± 1.6 (200.5; ***)
**Ile**	5.5 ± 0.3	7.9 ± 1.1 (144.7; *)	10.7 ± 0.7 (196.3; ***)
**Arg**	10.0 ± 1.1	12.2 ± 2.6 (122.7; ns)	17.9 ± 1.7 (180.1; ***)
**Asn**	76.4 ± 9.3	72.4 ± 5.4 (94.8; ns)	125.2 ± 33.7 (164.0; **)
**Lys**	6.6 ± 0.7	8.9 ± 0.4 (133.9; ns)	10.5 ± 0.9 (158.6; *)
**Gln**	213.6 ± 17.1	262.0 ± 13.9 (122.7; ns)	317.9 ± 52.1 (148.8; *)
**Phe**	6.3 ± 0.2	7.5 ± 0.8 (118.6; ns)	9.3 ± 0.4 (146.6; ns)
**Tyr**	1.5 ± 0.1	1.9 ± 0.1 (124.9; ns)	2.1 ± 0.1 (139.2; ns)
**Pro**	31.5 ± 3.2	33.7 ± 1.9 (107.0; ns)	42.4 ± 5.1 (134.5; ns
**Thr**	58.9 ± 3.7	68.1 ± 4.0 (115.5; ns)	77.9 ± 9.1 (132.2; ns)
**Glu**	652.1 ± 32.9	730.3 ± 22.2 (112.0; ns)	805.7 ± 90.0 (123.6; ns)
**Asp**	199.6 ± 15.0	200.9 ± 1.4 (100.6; ns)	243.3 ± 37.3 (121.9; ns)
**Met**	1.5 ± 0.1	1.4 ± 0.1 (97.3; ns)	1.7 ± 0.1 (116.7; ns)
**His**	13.5 ± 2.2	12.7 ± 1.2 (94.1; ns)	13.3 ± 0.9 (99.0; ns)
**Keto acid**	**amount of keto acid (pmol (mg FW)$^{-1}$); mean ± SEM (% compared to WT; significance level)**		
**KIC**	0.3 ± 0.03	3.5 ± 0.1 (1396.7 ± 45.8; ***)	3.8 ± 0.6 (1525.3 ± 238.2; ***)
**KIV**	0.4 ± 0.1	2.1 ± 0.1 (534.7 ± 25.7; ***)	2.9 ± 0.5 (731.5 ± 136.2; ***)
**KMV**	0.1 ± 0.03	0.6 ± 0.1 (436.9 ± 39.6; ***)	0.9 ± 0.1 (636.4 ± 82.7; ***)

Supplemental Table S2. Fe-S cluster containing subunits of complexes of the mETC with the estimated copy numbers in mitochondria isolated from heterotrophic Arabidopsis cell culture.

**accession number according to** (Przybyla-Toscano et al., 2021)	**gene name**	**estimated copy number according to** (Fuchs et al., 2020)	**Fe-S cluster**
complex I subunits			
At1g16700	TYKY	121	[Fe_4S_4]
At1g79010	TYKY isoform	2770	[Fe_4S_4]
At5g11770	PSST (20-kDa)	3492	[Fe_4S_4]
At4g02580	24-kDa	4683	[Fe_2S_2]
At5g08530	51-kDa	6131	[Fe_4S_4]
At5g37510	75-kDa	5521	[Fe_2S_2], [Fe_4S_4]
complex II subunits			
At3g27380	SDH2-1	1002	[Fe_2S_2], [Fe_3S_4], [Fe_4S_4]
At5g40650	SDH2-2	3441	[Fe_2S_2], [Fe_3S_4], [Fe_4S_4]
At5g65165	SDH2-3	-	[Fe_2S_2], [Fe_3S_4], [Fe_4S_4]
complex III subunits			
At5g13430; At5g13440	Ubiquinol-cytochrome C reductase iron-sulfur subunit	6657	Rieske, [Fe_2S_2]
